# Farnesoid X receptor antagonizes Wnt/β-catenin signaling in colorectal tumorigenesis

**DOI:** 10.1038/s41419-020-02819-w

**Published:** 2020-08-17

**Authors:** Junhui Yu, Shan Li, Jing Guo, Zhengshui Xu, Jianbao Zheng, Xuejun Sun

**Affiliations:** 1 Department of General Surgery, First Affiliated Hospital of Xi’an Jiaotong University, Xi’an, 710061 Shaanxi Province PR China; 2 Department of Reproductive Medicine, First Affiliated Hospital of Xi’an Jiaotong University, Xi’an, 710061 Shaanxi Province PR China

**Keywords:** Colon cancer, Oncogenes, Diagnostic markers

## Abstract

Farnesoid X receptor (FXR, encoded by NR1H4), a critical regulator of bile acid homeostasis, is widely implicated in human tumorigenesis. However, the functional role of FXR in colorectal cancer (CRC) and the precise molecular mechanism remain unclear. In this study, we demonstrated that FXR expression was downregulated in colon cancer tissues and decreased expression of FXR predicted a poor prognosis. Knockdown of FXR promoted colon cancer cell growth and invasion in vitro, and facilitated xenograft tumor formation and distant metastasis in vivo, whereas ectopic expression of FXR had the reserved change. Mechanistic studies indicated that FXR exerted its tumor suppressor functions by antagonizing Wnt/β-catenin signaling. Furthermore, we identified an FXR/β-catenin interaction in colon cancer cells. The FXR/β-catenin interaction impaired β-catenin/TCF4 complex formation. In addition, our study suggested a reciprocal relationship between FXR and β-catenin, since loss of β-catenin increased the transcriptional activation of SHP by FXR. Altogether, these data indicated that FXR functions a tumor-suppressor role in CRC by antagonizing Wnt/β-catenin signaling.

## Introduction

Colorectal cancer (CRC) ranks the second leading cause of cancer-related death^1^. Globally, ~1,800,000 new cases are diagnosed as CRC every year. With the change in lifestyle, including high-fat diets (HFDs), tobacco use and less or lack of exercise, the incidence of CRC has increased rapidly in developing countries^2,3^. Although great progress has been achieved in multimodality therapy of CRC, the prognosis of late-stage CRC is still unsatisfactory due to distant metastasis and relapse^4,5^. The molecular pathogenesis of CRC is considered a multistep and consecutive process with the accumulation of various aberrant genetic and epigenetic variations^6,7^. Dissecting the precise mechanisms of colorectal tumorigenesis is crucial for developing better prognostic and therapeutic strategies.

Canonical Wnt/β-catenin signaling plays essential roles in embryonic development and maintaining gut homeostasis^8^. Persistent activation of Wnt signaling featured by nuclear accumulation of β-catenin is an early event of colorectal tumorigenesis^9^. Wnt-related targets, including c-Myc, cyclin D1, MMP-7, and VEGF, are critical contributors to tumor cell proliferation, invasion, and migratory potential^10–12^. Epithelial-mesenchymal transition (EMT) is a biological process that involves the malignant transformation of epithelial cells with a loss of an epithelial and gain of a mesenchyme-like phenotype^13^. EMT plays a significant role in tumor progression via endowing tumor cells with the potential for invasive and metastatic growth. In invasive regions, tumor cells undergoing EMT exhibit a strong accumulation of nuclear β-catenin^14^. Recent study further demonstrates a direct link between β-catenin and EMT by identifying slug, a strong inducer of EMT, as the target of β-catenin^15^. These data strongly indicate that Wnt signaling participated in EMT process.

Bile acids are widely involved in the pathogenesis of human malignancies, including hepatocellular carcinoma (HCC)^16,17^, gastric cancer^18^, esophageal cancer^19,20^, and pancreatic cancer^21,22^. Exposure to elevated fecal bile acids is associated with the occurrence of colon cancer^23,24^. The farnesoid X receptor (FXR, encoded by NR1H4), a nuclear receptor of bile acids, is widely present in the gastrointestinal tract and liver^25,26^. In addition to its essential role in regulating bile acid homeostasis^27^, accumulating evidence supports a critical role of FXR in human tumorigenesis^28,29^. Reduced FXR at the mRNA level is found in colon polyps and is even more remarkable in CRC^30^. Restoration of FXR has been shown to suppress abnormal intestinal cell growth and curtail CRC progression^31^. However, the functional role of FXR in CRC and the precise molecular mechanism remain to be further elucidated.

In this study, we aim to investigate the correlation between FXR and Wnt/β-catenin signaling during colorectal tumorigenesis

## Materials and methods

### Clinical samples and cell cultures

One hundred and twenty-three human colon cancer tissues were obtained from patient diagnosed with colon cancer and received surgery at the First Affiliated Hospital of Xi’an Jiaotong University from January 2011 to May 2013. No patient had received preoperative chemotherapy or radiotherapy. The procedure of this study was approved by the institutional review board of the First Affiliated Hospital of Xi’an Jiaotong University. Informed consent was obtained by all participants.

Human colon cancer cells HT-29, Caco-2, HCT116, RKO, SW480, and Lovo were maintained in DMEM medium (Corning, New York, NY, USA) supplemented with 10% FBS (Hyclone, Logan, UT, USA) at 5% CO^2^ at 37 °C.

### Lentiviral vectors and transfection

The phU6-EGFP-shRNA-FXR, β-catenin, and SHP lentiviral vectors and their control vectors were used to inhibit FXR, β-catenin, and SHP expression, while the pUbi-EGFP- FXR, β-catenin, and SHP lentiviral vectors and their control vectors were used to increase FXR, β-catenin, and SHP expression. All transfections were conducted in accordance with the manufacturer’s protocol. All the lentiviral vectors were constructed and prepared by GeneChem Co., Ltd. (Shanghai, China)

### CCK8, colony formation and cell cycle assays

CCK8 assays were performed as described previously^32^. For colony formation assay, three hundred cells were seeded and cultured for 14 days. Colonies (≥50 cells/colony) were counted. Cell cycle distributions were evaluated by flow cytometry as previously described^32^. Each experiment was performed in triplicate.

### Wound-healing assays

Cells were cultured in six-well plates until confluent. Then, three artificial vertical lines were created with pipette tips (10 µL) in each well. The wells were washed with phosphate-buffered saline (PBS) to remove cell debris. The cells were then cultured for an additional 48 h. The scratch lines were imaged under a microscope, and the scratch distances were measured. Each experiment was performed in triplicate.

### Transwell assays

Cell migration and invasion were measured by using Transwell plates (Corning, New York, NY, USA) with or without Matrigel (BD, Franklin Lakes, NJ, USA). In both assays, the lower chamber was filled with 600 μl of DMEM medium containing 20% FBS. The upper chamber filters were pre-coated with 50 µL of Matrigel and plated at 10 × 10^4^ cells per upper chamber. The cells were incubated at 37 °C for 48 h. After incubation, non-migratory cells on the upper surface of the Transwell inserts were removed by washing with fresh PBS. The invading cells on the underside of the membrane were fixed with 4% paraformaldehyde and stained with 1% crystal violet. The number of cells was counted in three randomly selected fields of fixed cells under an inverted microscope. Each experiment was performed in triplicate.

### Nude mouse xenograft and lung metastasis models

All animal experiments were performed in accordance with the institutional guidelines, and were approved by the Laboratory Animal Center of Xi’an Jiaotong University. The 5-week-old female BALB/c-nude mice were purchased from Shanghai SLAC Laboratory Animal Co., Ltd. (Shanghai, China). The mice were injected with 5 × 10^6^ cells into the right flanks to establish xenograft tumor model. Tumor size was were monitored using callipers every 3 days, and the tumor volume was calculated according to the formula (length × width^2^ × 0.5). At the end of the experiment, the mice were killed and the xenograft tumors were isolated and weighted. Lung metastasis models were established via tail vein injections into each nude mouse. The weight of the nude mice was monitored every 3 days. At 60 days post-injection, the mice were killed by cervical dislocation, the lungs were excised, and the number of tumor nodules in the lung was recorded.

### RNA isolation and real-time PCR

RNA isolation, complementary DNA (cDNA) synthesized, and real-time PCR were performed as described previously^32^. The sequences of primers were summarized in Supplementary Table 1. Each experiment was performed in triplicate.

### Immunohistochemistry

The protocol was performed as previously described^32^. The extent of Ki67- and active caspase-3-stained cells was divided into 4 score ranks: 0–5% (0), 6–25% (1), 26–50% (2), 51–75% (3), and 76–100% (4). The staining intensity was divided into 4 score ranks: negative (0), light brown (1), brown (2), and dark brown (3). The immunoreactivity scores (IRSs) = extent score × intensity score. An IRS of ≤3 was defined as negative, and a score of >3 was defined as positive.

### Preparation of nuclear extracts

Nuclear extract was prepared with the protocol in the the Nuclear Extraction Kit (abcam, Cambridge, MA, USA). The nuclear protein was quantified and used for downstream applications.

### Total protein extraction and western blotting analysis

The detailed protocol was performed as described previously^32^. The antibody information was presented in Supplementary Table 2. Each experiment was performed in triplicate.

### Immunofluorescence (IF)

The cells were fixed with 4% paraformaldehyde for 20 min and permeabilized with 0.2% Triton X-100 for 10 min. After blocking with 5% bovine serum albumin (BSA) for 30 min at room temperature, the cells were incubated at 4 °C overnight with primary antibodies against E-cadherin and vimentin (1:100 dilution). The dishes were washed three times with PBS for 10 min each and then incubated with Alexa Fluor 594-conjugated secondary antibodies (1:400 dilution, Invitrogen, Carlsbad, CA, USA) for 1 h at room temperature. The nuclei were stained with DAPI (10 mg/ml) for 10 min. The samples were examined via microscopy (Leica Microsystems, Heidelberg, Germany) to analyze the expression of E-cadherin and vimentin.

### Luciferase reporter assay

Fragments of the SHP 5′-flanking sequence were amplified by PCR using special primers (Table S1) and cloned into the luciferase reporter vector pGL3.0-Basic (Promega, Madison, WI, USA) to generate SHP promoter reporter constructs (wild-type SHP promoter, SHP-WT promoter). Mutagenesis of FXR binding site in SHP promoter (SHP-mFXR promoter) was performed using a site-directed mutagenesis kit (Takara Biotechnology Co., Ltd.). The detailed protocol was performed as described previously^32^. Each experiment was performed in triplicate.

### Co-immunoprecipitation assay

The cells were washed three times with ice-cold PBS and harvested at 4 °C in immunoprecipitation lysis buffer. Co-immunoprecipitation (co-IP) assay was then performed as described previously^33^. Each experiment was performed in triplicate.

### Quantitative chromatin immunoprecipitation

Cells were subjected to ChIP using the EZ-ChIP Kit (Millipore, Bedford, MA, USA)。

The detailed protocol was performed as described previously^32^. Real-time PCR was conducted to amplify the regions of DNA fragments by using special primers (Supplementary Table 1). Each experiment was performed in triplicate.

### Microarrays and gene expression analysis

Three HT-29-shCtrl cells and three HT-29-shFXR cells were used for microarrays. Total RNA was extracted using TRIzol reagent (Invitrogen, Carlsbad, CA, USA). An Affymetrix PrimeView Human Gene Expression Array was used to investigate the changes in transcriptional profiles. The experiment was performed based on the manufacturer’s standard protocols. Genes with ≥1.5-fold change between two groups were identified as differentially expressed genes.

### Statistical analysis

Each experiment was repeated three times. Data are presented as the mean ± SD. The Student’s *t* test, one-way ANOVA or *χ*^2^ test was conducted to compare the differences among the groups. Correlations were analyzed by using Pearson linear-regression analysis. OS and RFS rates were plotted using the Kaplan–Meier method and compared with log-rank test. Multivariate statistical analysis was performed using a Cox regression model. Statistical analyses were performed with SPSS 18.0 software (SPSS Inc., Chicago, IL, USA). *P* < 0.05 was considered statistically significant.

## Results

### FXR is expressed at low levels in colon cancer clinical samples and correlates with poor prognosis

**Fig. 1:**
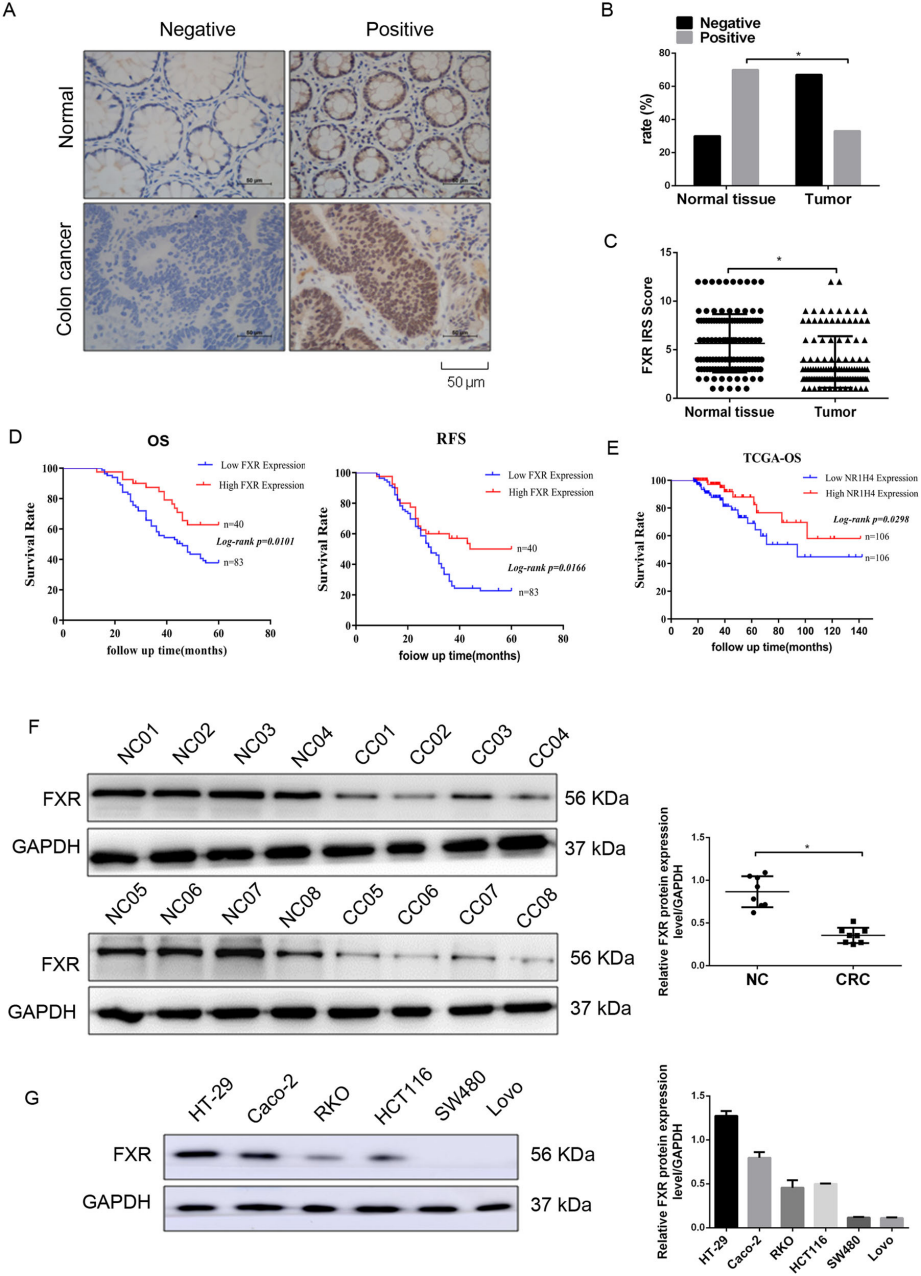
The expression of FXR in colon cancer tissue samples and normal tissue samples. **a** The expression of FXR in normal tissue samples and colon cancer tissue samples by immunohistochemistry (IHC) staining. **b** The positivity of FXR staining in normal tissue samples and colon cancer tissue samples. **c** The immunoreactivity score (IRS) of FXR staining in normal tissue samples and colon cancer tissue samples. **d** Kaplan–Meier representation of the overall survival and recurrence-free survival of the two groups of patients with high (*n* = 40, red line) or low (*n* = 83, blue line) FXR expression in colon cancer tissues. **e** Data in TCGA database showed the overall survival of the two groups of patients with high (red line) or low (blue line) FXR expression in colon cancer tissues. **f** Western blotting bands for FXR expression in normal tissue samples and colon cancer tissue samples. **g** Western blotting bands for FXR expression in six colon cancer cells. All data are the mean ± SD of three independent experiments. *P < 0.05.

To determine whether FXR correlates with CRC development and progression, we first employed immunohistochemistry (IHC) assay to detect the expression of FXR in 123 colon cancer tissues and paired normal tissues. FXR immunostaining was seen in the nuclei of colonic cells (Fig. 1a). The rate of positive FXR staining was decreased from 67.5% (83/123) in normal tissues to 32.5% (40/123) in colon cancer tissues (Fig. 1b). Moreover, colon cancer tissues displayed a low immunoreactivity score (IRS) of FXR staining in relative to normal tissues. The correlation between FXR and patient clinicopathological characteristics was further analyzed. Our study found that FXR expression was negatively correlated with tumor size, T stages, lymph node metastasis, and TNM stages (Supplementary Table 3). Kaplan–Meier analysis showed that patients with low FXR-expressed tumors had shorter overall survival (OS) times and shorter recurrence-free survival (RFS) times than those with high FXR-expressed tumors (Fig. 1d). Univariate analyses indicated that FXR expression was negatively correlated with OS and RFS, although FXR was not validated as an independent predictor of OS and RFS by multivariate analyses (Supplementary Tables 4 and 5). Analyses of colon cancer data from the TCGA database also supported a strong relationship between diminished FXR and poor overall survival in patients with colon cancer (Fig. 1e).

Next, western blotting analysis was conducted to evaluate the expression of FXR in eight colon cancer tissues and paired normal tissues (Fig. 1f). The results showed that tumor tissues exhibited reduced FXR expression levels compared with normal tissues (Fig. 1g). Finally, the expression of FXR in six colon cancer cell lines was investigated (Fig. 1h). FXR was highly expressed in highly or moderately differentiated HT-29 and Caco-2 cells. However, in poorly differentiated (HCT116 and RKO) or undifferentiated (SW480 and Lovo) colon cancer cells, the levels of FXR were at a low level or not expressed. Taken together, we conclude that FXR expression is reduced in colon cancer tissues and decreased FXR expression correlates with poor prognosis.

### FXR inhibits tumorigenic properties of colon cancer cells

**Fig. 2:**
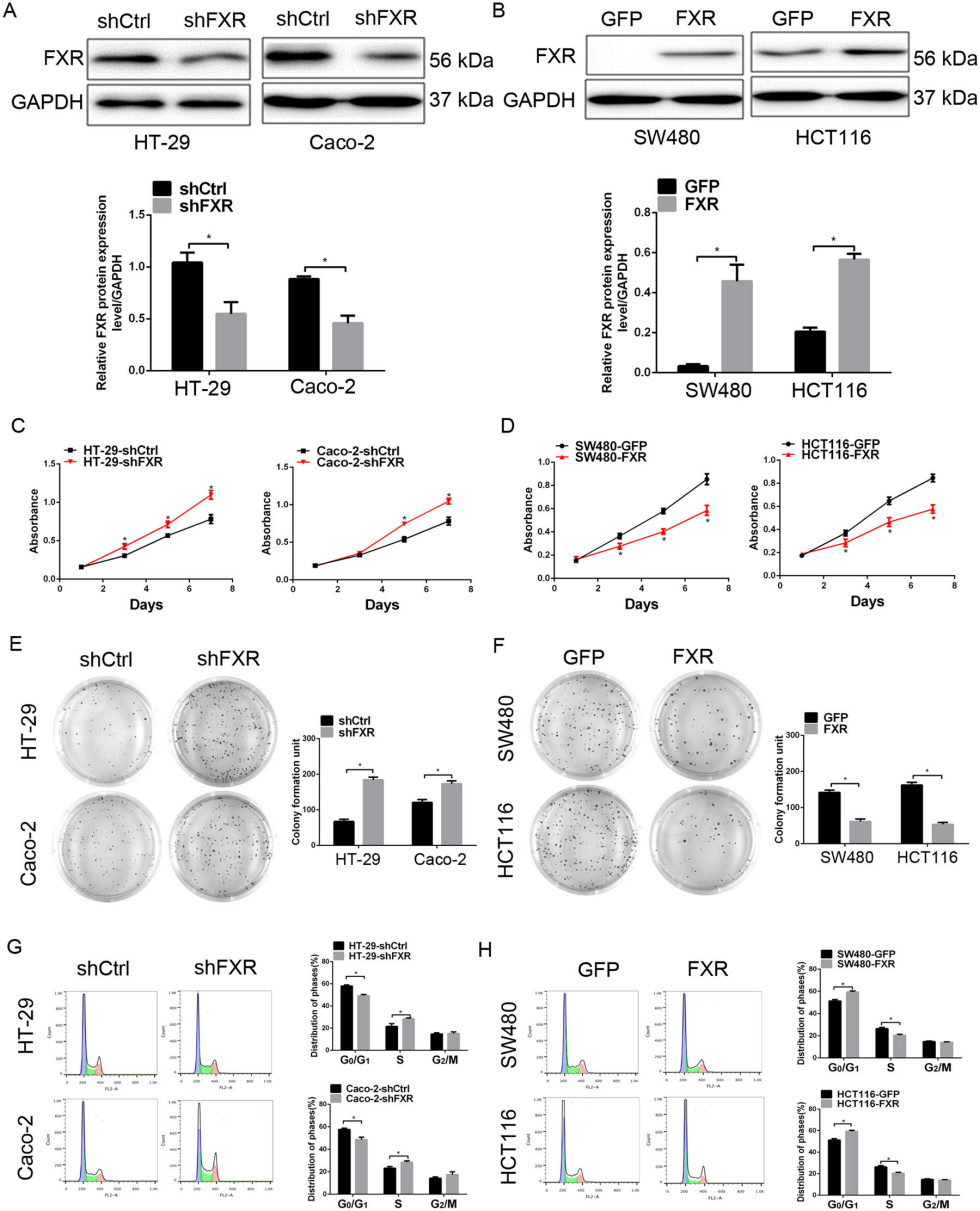
FXR inhibits tumorigenic properties of colon cancer cells. **a**, **b** FXR expression in FXR-knockdown HT-29 and Caco-2 cells (**a**) or FXR-overexpressing SW480 and HCT116 cells (**b**) detected by western blotting analysis. **c**, **d** The effect of FXR knockdown (**c**) or overexpression (**d**) on the viability of colon cancer cells detected by CCK8 assays. **e**, **f** The effect of FXR knockdown (**e**) or overexpression (**f**) on colony formation of colon cancer cells. **g**, **h** The effect of FXR knockdown (**g**) or overexpression (**h**) on cell cycle distribution of colon cancer cells. All data are the mean ± SD of three independent experiments. *P < 0.05.

To further gain insight into the impact of FXR in colorectal tumorigenesis, a series of in vitro experiments were performed in colon cancer cells with gain-of-function and loss-of-function of FXR. Knockdown of FXR in HT-29 and Caco-2 cells, and ectopic expression of FXR in SW480 and HCT116 cells were validated by western blotting analysis (Fig. 2a, b).

CCK8 assays were employed to assess the effect of modulating FXR expression on the viability of colon cancer cells. Our data indicated that knockdown of FXR in HT-29 and Caco-2 cells resulted in an enhanced cell viability (Fig. 2c), whereas ectopic expression of FXR in SW480 and HCT116 cells had the reserved change (Fig. 2d). The inhibitory effect of FXR on tumor cell growth was further verified by colony-formation assay, in which knockdown or ectopic expression of FXR promoted or inhibited the colony-formation ability of colon cancer cells (Fig. 2e, f), respectively. As cell proliferation regulation was observed after modulation of FXR, cell cycle distribution was detected by flow cytometry assay. Knockdown of FXR resulted in a marked decrease of cells in the G0/G1 phase with an accumulation of cells the S phase (Fig. 2g). FXR overexpression experiment had the opposite change (Fig. 2h). However, modulation of FXR expression had no significant impact on the accumulation of cells in the G2 phase.

We next conducted xenograft mouse model to assess in vivo tumor-suppressor role of FXR. The xenograft tumors in the FXR-knockdown group showed a decline in growth rate in relative to the control group (Supplementary Fig. 1a, b). Moreover, mean weight of xenograft tumors in the FXR-knockdown group is lighter than that in the control group (Supplementary Fig. 1a, b). FXR-overexpressing group displayed the opposite change (Supplementary Fig. 1c, d). Ki67 was a well-known maker evaluating cellular proliferation. Thus, we detected Ki67 expression in xenograft tumors by using IHC staining. Enhanced Ki67 staining of the xenograft tumors was observed in the FXR-knockdown group in relative to the control group (Supplementary Fig. 1e). Conversely, the xenograft tumors in the FXR-overexpressing group had the opposite change (Supplementary Fig. 1f). Collectively, these data supported a tumor suppressor role of FXR in colon cancer cells.

### FXR inhibits colon cancer cell invasion and metastasis in vitro and vivo

Considering that tumor metastasis is the leading cause of cancer-related death in CRC, we thus aimed to evaluate the impact of FXR on the invasive and migratory abilities of colon cancer cells. Wound-healing scratch assays showed that knockdown of FXR in HT-29 and Caco-2 cells led to an increase in the percentage of wound healing (Supplementary Fig. 2a, b). Conversely, ectopic expression of FXR in SW480 and HCT116 cells had the reserved change (Supplementary Fig. 2c, d). The Transwell assays showed that the number of invasive HT-29-shFXR and Caco-2-shFXR cells was greater than the number of invasive control cells (Supplementary Fig. 2e, g), whereas the number of invasive SW480-FXR and HCT116-FXR cells was less than the number of invasive control cells (Supplementary Fig. 2f, h).

**Fig. 3:**
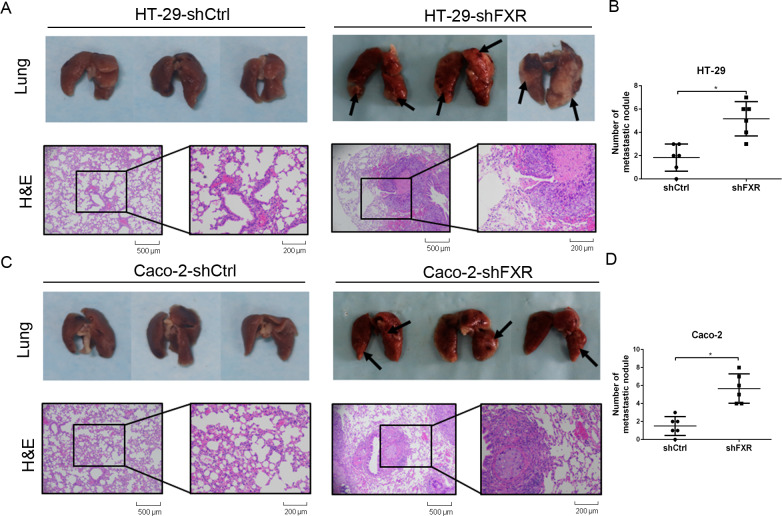
Knockdown of FXR promotes the lung metastasis of colon cancer cells in vivo. Lung metastasis models of colon cancer were generated in BALB/c-nude mice with FXR-knockdown HT-29 and Caco-2 cells via tail vein injection. **a**, **c** The metastases in lung were shown for HT-29-shFXR (**a**) and Caco-2-shFXR (**c**) group and their control group identified by H&E staining. **b**, **d** Average number of tumor nodules in lung metastasis in the HT-29-shFXR (**b**) and Caco-2-shFXR (**d**) group and their control group. *P < 0.05.

To determine whether FXR affects colon cancer cell metastasis in vivo, colon cancer lung metastasis models via tail vein injection were generated in BALB/c-nude mice using HT-29 and Caco-2 cells with stably FXR knockdown. The metastatic tumor nodules of the FXR-knockdown group and the control group were counted under microscopy by H&E staining (Fig. 3a, c). The average number of tumor nodules in lung metastasis in HT-29-shFXR and Caco-2-shFXR groups was greater than that in the control groups (Fig. 3b, d). Altogether, these data suggested that FXR inhibits colon cancer cells invasion and metastasis.

### FXR inhibits EMT in colon cancer cells

The presence of tumor cells that have undergone EMT is considered to be an important event during the early stage of cancer metastasis^34^. Whether EMT is involved in the mechanism of FXR-mediated inhibition of colorectal tumorigenesis was investigated. Our study indicated that knockdown of FXR in HT-29 and Caco-2 cells resulted in a promotion in the levels of slug, snail, vimentin, fibronectin, and MMP-9 with a reduction in the levels of E-cadherin and ZO-1 (Supplementary Fig. 3a, c). Conversely, ectopic expression of FXR had the opposite effect (Supplementary Fig. 3b, d).

Furthermore, the results from IF assay indicated that HT-29-shFXR and Caco-2-shFXR cells showed a decrease in E-cadherin staining (Supplementary Fig. 4a, b) and an increase vimentin staining (Supplementary Fig. 4c, d) in relative to the control cells. IHC assay showed the lung metastatic tumor tissues formed in the FXR-knockdown group demonstrated a weaker E-cadherin staining and a much stronger vimentin-staining than those formed in the control group (Supplementary Fig. 4e, f). Altogether, these findings supported a suppressive effect of FXR on EMT in CRC.

### FXR inhibits colorectal tumorigenesis by antagonizing Wnt/β-catenin signaling

**Fig. 4:**
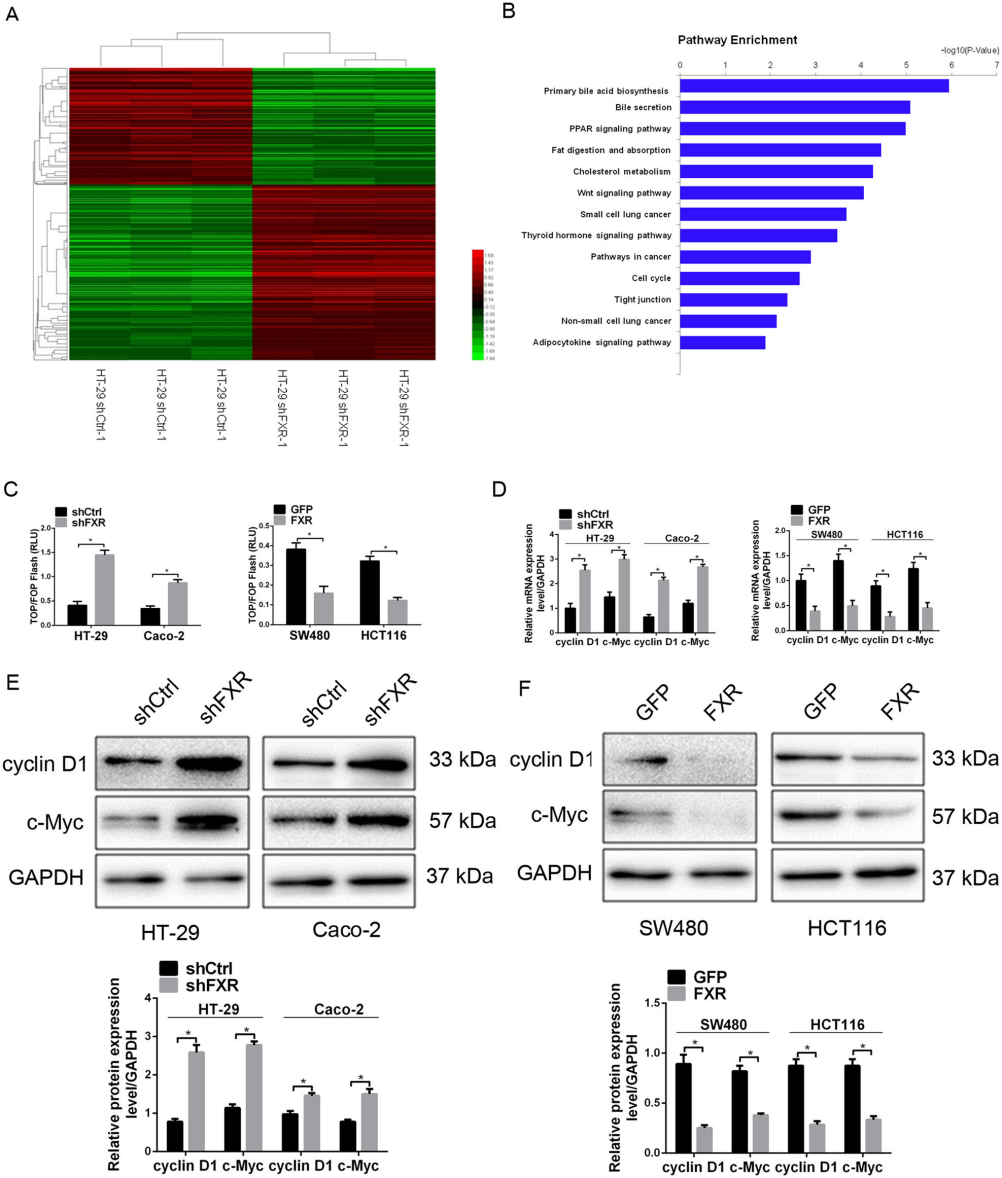
FXR suppresses the activity of Wnt/β-catenin signaling in colorectal tumorigenesis. **a** Hierarchical clustering of genes that were significantly and differentially expressed in HT-29-shFXR cells and control cells. Data were log2 normalized. **b** Pathway analysis of genes that were significantly and differentially expressed in HT-29-shFXR cells and control cells using KEGG database. **c** The effect of FXR knockdown or overexpression on the luciferase activities of TOP/FOP-Flash reporter plasmid. **d** The effect of FXR knockdown or overexpression on the mRNA levels of cyclin D1 and c-Myc in colon cancer cells detected by real-time PCR. **e**, **f** The effect of FXR knockdown (**e**) or overexpression (**f**) on the protein levels of cyclin D1 and c-Myc in colon cancer cells detected by western blotting analysis. All data are the mean ± SD of three independent experiments. *P < 0.05.

We further explore the mechanism of FXR-mediated tumorigenicity and EMT inhibition by using microarray analyses of HT-29-shFXR cells and the control cells. Hierarchical clustering analysis showed that knockdown of FXR led to a difference in gene expression profile with the upregulation of 310 genes and the downregulation of 206 genes (Fig. 4a). Pathway analysis suggested that multiple signaling pathway might participate in the tumor-promoting mechanism of FXR knockdown (Fig. 4b). Among them, Wnt/β-catenin pathway aroused our interest, as it plays a critical role in promoting EMT, stemness properties and tumorigenicity^35^. In addition, previous studies revealed that FXR deficiency in mice led to increased colon and liver cell proliferation, accompanied by an upregulation of β-catenin^36,37^. Analysis of Wnt/β-catenin signaling indicated that c-Myc and cyclin D1, two direct target genes, were upregulated in HT-29-shFXR cells in relative to HT-29-shCtrl cells (data not shown). Hence, Wnt/β–catenin signaling was chosen for further research.

To further validate the impact of FXR on the activity of Wnt/β-catenin signaling in colon cancer cells, we first performed a TOP/FOP-Flash luciferase reporter assay. Knockdown of FXR in HT-29 and SW480 cells elevated the luciferase intensities compared with the control (Fig. 4c). Moreover, the expression of c-Myc and cyclin D1 at both the mRNA and protein levels was elevated in response to FXR knockdown (Fig. 4d, e). FXR overexpression experiment had the opposite change (Fig. 4c, d, f).

Furthermore, XAV-939, a selective inhibitor of Wnt signaling, was adopted to block activated Wnt signaling in FXR-knockdown cells. As expected, XAV-939 treatment attenuated the proliferative and invasive abilities inhibited by FXR knockdown (Supplementary Fig. 5a–f). Consistent with the observations above, XAV-939 treatment reduced the protein levels of vimentin, fibronectin, MMP-9, snail, slug, cyclin D1, and c-Myc but increased the level of E-cadherin and ZO-1 (Supplementary Fig. 6a–d). These data demonstrate that FXR inhibits colorectal tumorigenesis and EMT induction, which may be attributed to its suppression of Wnt signaling.

### FXR functions as a repressor of Wnt/β-catenin signaling by interacting with β-catenin in colon cancer cells

**Fig. 5:**
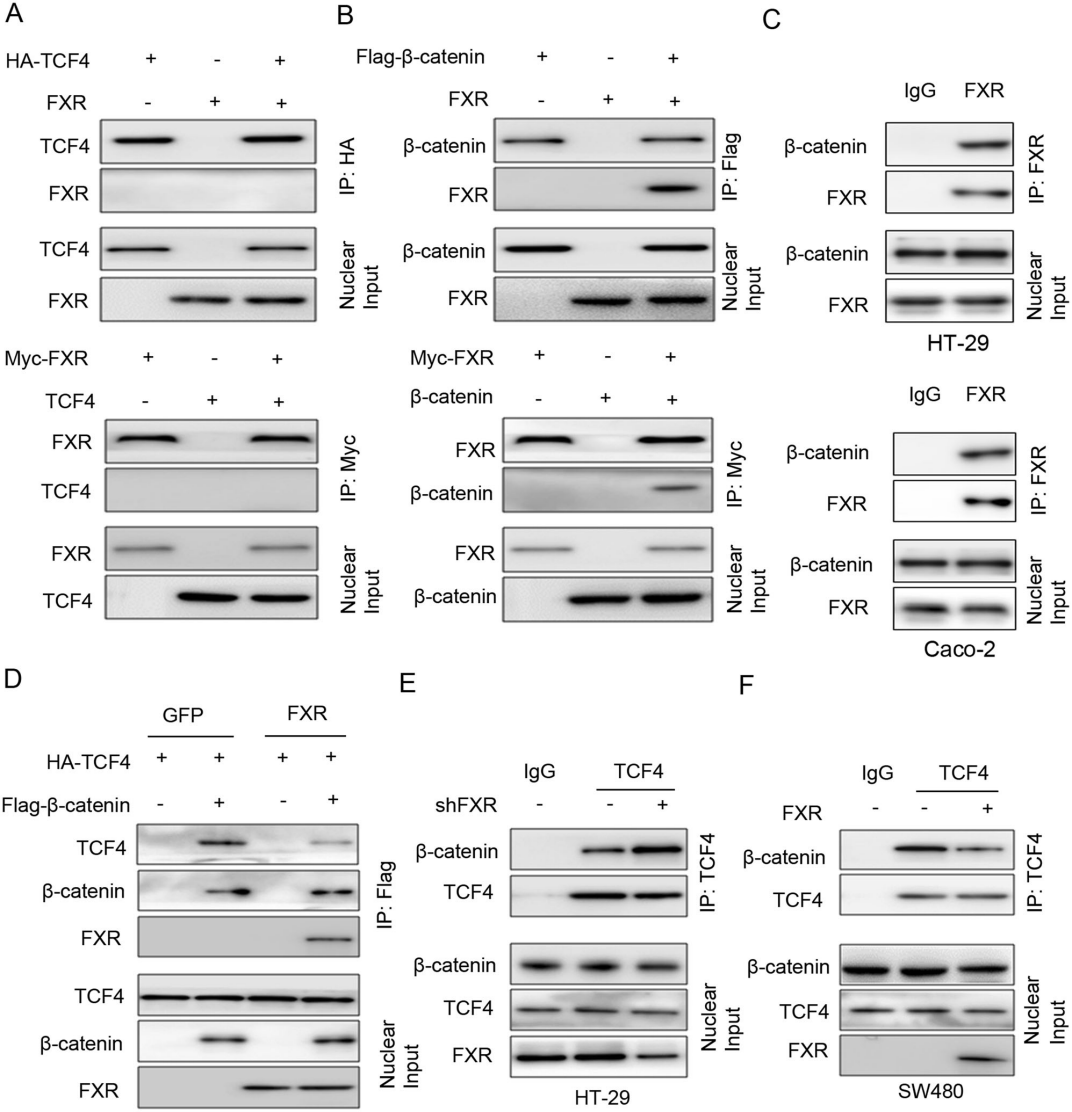
FXR functions as a repressor of Wnt/β-catenin signaling by interacting with β-catenin in colon cancer cells. **a** Co-immunoprecipitation showed the physical interaction between FXR and TCF4 in HEK293 cells co-expressing HA-tagged TCF4 and Myc-tagged FXR. The cell lysates were subjected to IP with an anti-HA or Myc antibody. **b** Co-immunoprecipitation showed the physical interaction between FXR and β-catenin in HEK293 cells co-expressing Flag-β-catenin and Myc-tagged FXR. The cell lysates were subjected to IP with an anti-Flag or Myc antibody. **c** Co-immunoprecipitation showed the physical interaction between endogenous FXR and β-catenin was also observed in HT-29 and Caco-2 cells. The cell lysates were subjected to IP with an anti-FXR. **d** Ectopic expression of FXR impaired the interaction of β-catenin and TCF4. Plasmids of HA-tagged-TCF4, Flag-tagged-β-catenin, and FXR-shRNA lentivirus were co-transfected into HEK293 cells. The cell lysates were subjected to IP with an anti-Flag antibody. **e** Knockdown of FXR enhanced the interaction of β-catenin and TCF4. HT-29 cells were infected with FXR-shRNA lentivirus or control lentivirus. The nuclear fractions were incubated with an anti-TCF4 antibody for the IP experiment. IgG was used as a negative control. **f** Ectopic expression of FXR enhanced the interaction of β-catenin and TCF4. SW480 cells were infected with FXR lentivirus or control lentivirus. The nuclear fractions were incubated with an anti-TCF4 antibody for the IP experiment.

In canonical Wnt signaling, elevated nuclear β-catenin is always observed^38^. However, our western blotting analysis showed no significant difference in nuclear β-catenin level between modified-FXR cells and the control cells (data not shown). Since FXR is a mainly located in the nucleus, we hypothesized that the FXR might affect the β-catenin/TCF complex^38^. First, the co-IP assay showed that FXR did not bind with TCF4 in the nucleus of HEK293 cells (Fig. 5a). Then, we questioned whether FXR could form a complex with β-catenin. FXR co-immunoprecipitated with β-catenin in HEK293 cells co-transfected with FXR and Flag-β-catenin (Fig. 5b). Moreover, reciprocal co-IP experiments were performed by co-transfecting Myc-FXR and β-catenin into HEK293 cells, which further support the interaction between FXR and β-catenin (Fig. 5b). An interaction between endogenous FXR and β-catenin was also observed in HT-29 and Caco-2 cells (Fig. 5c). Next, we attempted to assess that whether FXR abolished the stability of the β-catenin/TCF4 complex by forming a complex with β-catenin. The binding between exogenous β-catenin and exogenous TCF4 was impaired in HEK293 cells upon FXR overexpression (Fig. 5d). Moreover, knockdown of FXR in HT-29 cells retarded the binding between endogenous β-catenin and endogenous TCF4 (Fig. 5e), whereas ectopic expression of FXR in SW480 cells had the opposite effect (Fig. 5f). Altogether, these data indicated that FXR functions as a repressor of Wnt/β-catenin signaling by interacting with β-catenin. This interaction impaired β-catenin/TCF4 complex and subsequent transcriptional activity of Wnt-related target genes.

### FXR inhibits colorectal tumorigenesis by regulating SHP expression

**Fig. 6:**
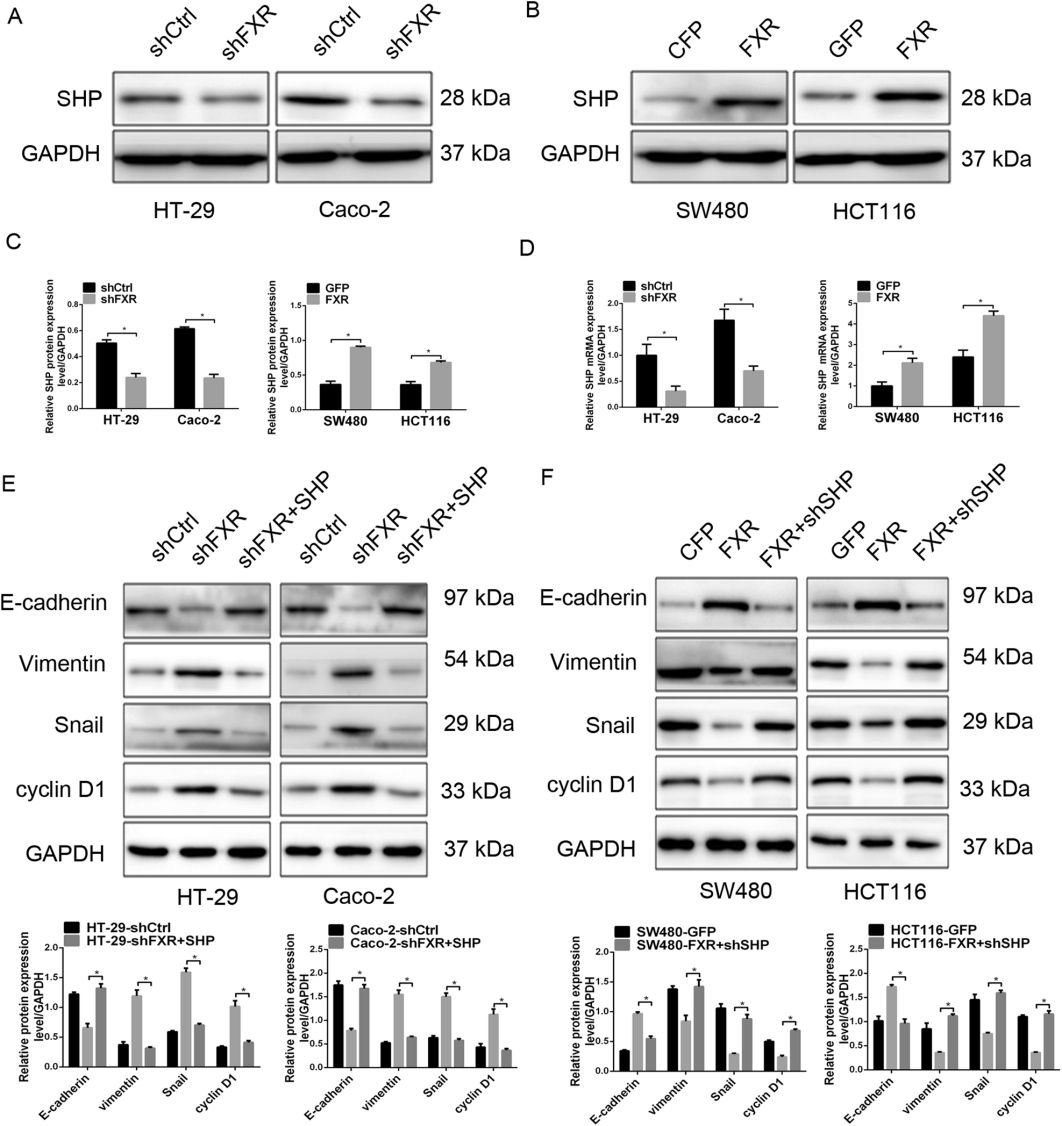
FXR inhibits colorectal tumorigenesis by regulating SHP expression. SHP expression in FXR-knockdown HT-29 and Caco-2 cells (**a**) or FXR-overexpressing SW480 and HCT116 cells (**b**) detected by western blotting analysis. **c** Quantitative analysis of SHP expression in FXR-knockdown HT-29 and Caco-2 cells or FXR-overexpressing SW480 and HCT116 cells. **d** SHP expression in FXR-knockdown HT-29 and Caco-2 cells or FXR-overexpressing SW480 and HCT116 cells detected by real-time PCR. **e** The effect of SHP overexpression on cell cycle- and EMT-related protein levels of FXR-knockdown HT-29 and Caco-2 cells detected by western blotting analysis. **f** The effect of SHP knockdown on cell cycle- and EMT-related protein levels of FXR-overexpressing SW480 and HCT116 cells detected by western blotting analysis. All data are the mean ± SD of three independent experiments. *P < 0.05.

Small heterodimer partner (SHP), the well-known target gene of FXR^39^, retards tumorigenesis by regulating cyclin D1 expression^40^. Microarray analyses revealed that SHP is downregulated in HT-29 cells upon FXR knockdown, which was validated by the results of real-time PCR and western blotting analysis (Fig. 6a–d). To further validate the involvement of SHP in FXR-mediated inhibition of colorectal tumorigenesis, we ectopically expressed or knocked down SHP in colon cancer cells. Ectopic expression of SHP attenuated the proliferative and invasive abilities of colon cancer cells enhanced by FXR knockdown (Supplementary Fig. 7a, c, e). Western blotting analysis indicated that the levels of cell cycle- and EMT-related proteins in HT-29-shFXR and Caco-2-shFXR cells was reversed upon ectopic expression of SHP (Fig. 6e). Conversely, knockdown of SHP significantly restored the tumor-inhibitory effect of FXR on SW480 and HCT116 cells (Supplementary Fig. 7b, d, f) as well as the levels of cell cycle- and EMT-related proteins (Fig. 6f). These findings further confirm that FXR-mediated colorectal tumorigenesis inhibition might be partly related to its transcriptional activation of SHP.

### Modulation of β-catenin impacts FXR transcriptional activation of SHP expression in colon cancer cells

**Fig. 7:**
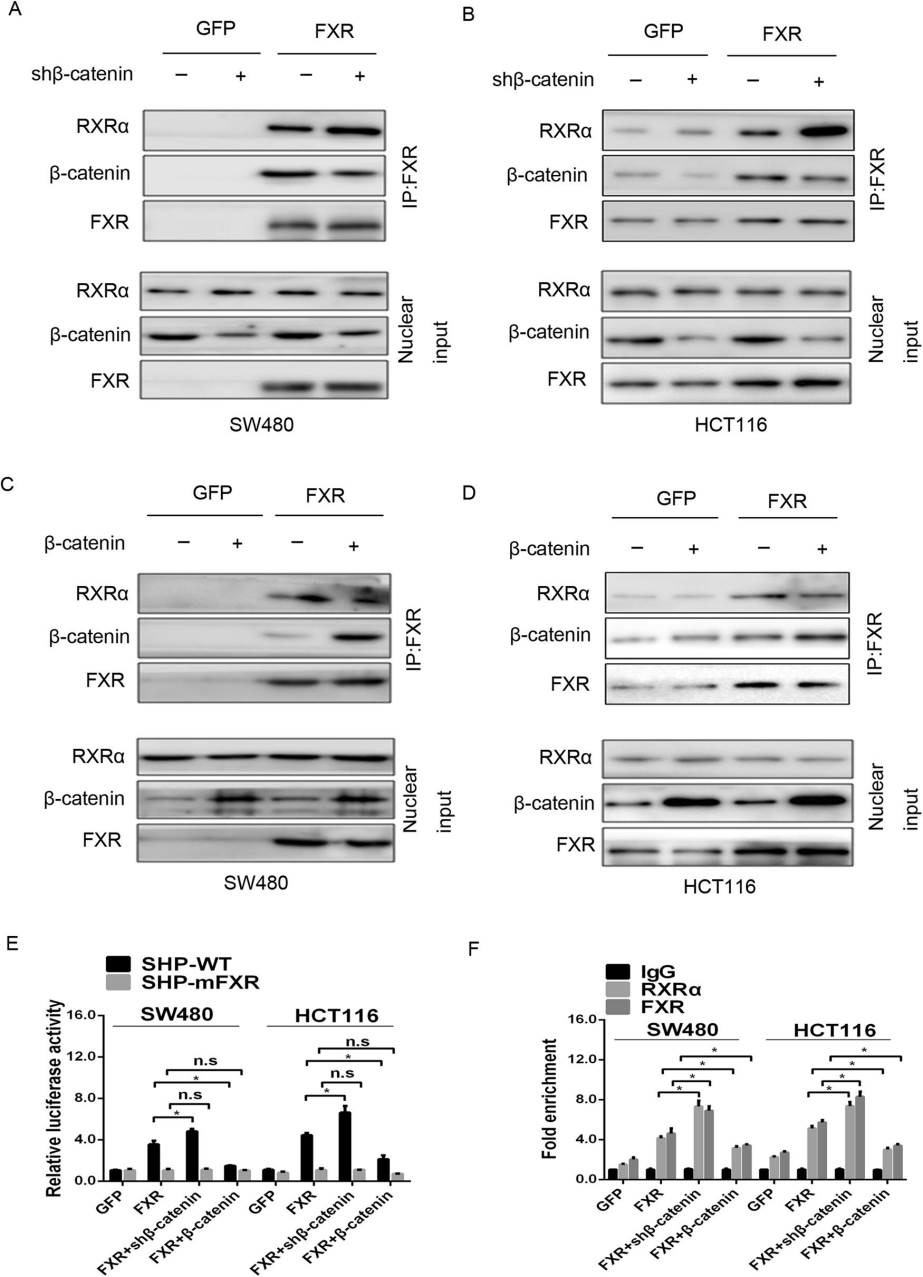
Modulating β-catenin expression affected the FXR/RXRα interaction in colon cancer cells. **a**, **b** Knockdown of β-catenin enhanced the FXR/RXRα interaction. SW480-FXR (**a**) and HCT116-FXR (**b**) cells and their control cells were infected with β-catenin-shRNA lentivirus or control lentivirus as indicated above for 48 h. The nuclear fractions were incubated with an anti-FXR antibody for the IP experiment. **c**, **d** Ectopic expression of β-catenin impaired the FXR/RXRα interaction. SW480-FXR (**c**) and HCT116-FXR (**d**) cells and their control cells were infected with β-catenin-overexpressing lentivirus or control lentivirus as indicated above for 48 h. The nuclear fractions were incubated with an anti-FXR antibody for the IP experiment. **e** Modulating β-catenin expression affected the binding of RXRα to SHP promoter. Wild type SHP promoter (SHP-WT) or a mutated SHP loss of FXR binding site (SHP-mFXR) and pRL-TK plasmid were transfected into HEK293 cells for 24 h. Luciferase activity was measured using cell lysates 24 h after transfection. **f** The effect of modulating β-catenin expression on the binding of FXR or RXRα protein to the SHP promoter detected by the qChIP assay. All data are the mean ± SD of three independent experiments. *P < 0.05.

To determine whether the FXR/β-catenin interaction affects FXR transcriptional activation of SHP expression, we first assessed the impact of modulating β-catenin on SHP expression. Knockdown of β-catenin in SW480-FXR and HCT116-FXR cells elevated the expression of SHP both at the mRNA and protein levels compared with the control (Supplementary Fig. 8a, c). Conversely, ectopic expression of β-catenin had the opposite effect (Supplementary Fig. 8b, d). FXR often regulate target genes transcription as a heterodimer with the retinoid X receptor (RXR)^41^. We then questioned whether modulation of β-catenin affected the FXR/RXRα complex. The co-IP assay showed that knockdown of β-catenin in SW480-FXR and HCT116-FXR cells resulted in a reduction in the FXR/β-catenin complex but a promotion in the FXR/RXRα complex (Fig. 7a, b), whereas ectopic expression of β-catenin had the reserved effect (Fig. 7c, d). Furthermore, knockdown of β-catenin in SW480-FXR and HCT116-FXR cells increased the luciferase activities of the SHP-WT promoter but not the luciferase activities of the SHP-mFXR promoter. Conversely, ectopic expression of β-catenin decreased the luciferase activities of the SHP-WT promoter but not the SHP-mFXR promoter (Fig. 7e).

Finally, a quantitative chromatin immunoprecipitation (qChIP) assay was employed to determine whether modulation of β-catenin affected the occupancy of FXR on the SHP promoter in vivo. Our data revealed that knockdown of β-catenin led to an enhancement of FXR binding to the SHP promoter in SW480-FXR and HCT116-FXR cells (Fig. 7f). Conversely, ectopic expression of β-catenin exerted an opposite effect. Interestingly, RXRα occupancy of the SHP promoter tended to mirror that of FXR, which is likely due to the occupancy of its binding partner FXR and not because of any direct association with β-catenin. These data indicate that the FXR/β-catenin complex antagonizes the FXR/RXRα complex and its transcriptional activity.

### Correlations among FXR and cell cycle- and EMT-related proteins in colon cancer tissues

Correlations among FXR and cell cycle- and EMT-related proteins were further explored in 30 colon cancer tissues by IHC assay (Supplementary Fig. 9a). Our study showed that the level of FXR positively correlated with that of SHP (Supplementary Fig. 9b) and E-cadherin (Supplementary Fig. 9c) and negatively correlated with that of vimentin, cyclin D1, and c-Myc expression (Supplementary Fig. 9d–f), further supporting the notion that FXR is a negative regulator of colorectal tumorigenesis.

## Discussion

Mounting epidemiological evidence indicates that HFDs, rich in carbohydrates and saturated fatty acids, are an acknowledged risk factor for CRC^42^. HFDs lead to commensurate increases in fecal bile acids, particularly lithocholic and deoxycholic acids, which are potent inducers of CRC^43^. FXR, a bile acid-activated nuclear receptor, plays an important role on oncogenic transformation. In this study, we demonstrated that FXR expression was significantly downregulated in colon cancer tissues and decreased FXR expression was negatively related to the location of tumor, lymph node metastasis, and TNM stage. High FXR expression is a strong and independent prognostic indicator in invasive breast carcinoma^44^. In colon cancer, low FXR expression was correlated with worse clinical outcome^45^. Our date concluded that reduced FXR expression was significantly associated with worse OS and RFS of patients with CRC, although FXR was not validated as an independent predictor of OS and RFS by multivariate analyses.

In vitro and in vivo assays further supported a tumor-suppressor role of FXR in CRC. Knockdown of FXR promoted colon cancer cells cell growth and invasion in vitro, and facilitated tumor formation and distant metastasis in vivo. These changes were the opposite of those seen in the FXR overexpression experiment and further validated the role of FXR in CRC progression. Consistent with our study, FXR-deficient mice exhibited an enhancement in intestinal cell proliferation with upregulation of cyclin D1 expression^36^. Moreover, our study demonstrated that knockdown of FXR in colon cancer cells induced EMT, accompanied by upregulation of Snail, Slug, vimentin, fibronectin, and MMP-9, and downregulation of E-cadherin and ZO-1, whereas ectopic expression of FXR had reversed change. In non-small-cell lung cancer, FXR functions as a proto-oncogene, promoting cell proliferation by directly transactivating cyclin D1^46^. In pancreatic cancer, increased FXR promotes cell invasive and migratory ability^47^. These studies indicate the tumor-specific contributions of FXR to the pathogenesis of different cancer types.

Previous study indicated that in FXR-deficient mice, increased activation of Wnt signaling was observed in spontaneous HCC^37^. The present study revealed that knockdown of FXR activated Wnt signaling, as evidenced by the high luciferase activity of the TOP/FOP-Flash reporter and the upregulation of Wnt signaling target genes. These changes were opposite to FXR overexpression experiment. Moreover, blockage of Wnt signaling by XAV-939 attenuated the tumor-suppressive effect of FXR knockdown on colon cancer cells. Activation of Wnt/β-catenin signaling is a strong inducer of EMT, as evidenced by the identification of β-catenin-regulated genes, such as fibronectin^48^, slug^15^, MMP-7^49^, and VEGF^50^. These genes, directly or indirectly involved in EMT, code for direct effectors of CRC progression. These data indicated that FXR-inhibited tumorigenicity and EMT in CRC could be attributed to the inactivation of Wnt/β-catenin signaling. In addition, activation of FXR abolished colon cancer cell growth by inhibiting EGFR/ERK signaling^51^. Intriguingly, a recent study revealed that FXR directly regulated MMP-7 expression by acting as a transcriptional repressor^52^. These data indicate that FXR exerts its tumor suppressor functions via distinct signaling pathways.

FXR has been widely considered a transcriptional factor functioning in cancer cells^53^. In the present study, the expression of SHP, a well-known target gene of FXR, was decreased or elevated upon FXR knockdown or FXR overexpression. SHP has also been shown to suppress tumor cell proliferation and invasion via transcriptional repression of cyclin D1 and Ccl2 expression^40,54^. Our study demonstrated that the tumor-suppressive effects of FXR could be partially attributed to FXR-mediated transcriptional activation of SHP, as ectopic expression of SHP impaired the proliferative and invasive potential of colon cancer cells.

The crosstalk between Wnt signaling and the nuclear receptors (NRs) has been highlighted in the field of cancer research^55^. Analyses of NR interactions with canonical Wnt signaling reveal two broad themes: Wnt/β-catenin modulation of NRs (theme I) and NR inhibition of the Wnt/β-catenin/TCF cascade (theme II). Glucocorticoid receptor (GR) could inhibit tumor proliferation by repressing cyclin D1 expression via targeting of the β-catenin/TCF complex^56^ (theme II); and β-catenin acts as a coactivator of androgen receptor (AR) transcription and promotes cell proliferation and prostate pathogenesis^57^ (theme I). Our study revealed that a nontranscriptional mechanism of FXR that FXR forms a complex with β-catenin and subsequently disturbs the transcriptional activity of the β-catenin/TCF complex in colon cancer lines (theme II). FXR appears to regulate β-catenin activity without affecting β-catenin localization and protein stability, since modulation of FXR expression did not affect the translocation of β-catenin (data not shown). On the other hand, our study found that modulation of β-catenin affected the transcriptional activation of SHP by FXR, indicating that β-catenin negatively regulates FXR activity through direct binding (theme I). Based on these observations, we propose a model in which an event initiated in tumor cells activates Wnt signaling at the early phase of colorectal tumorigenesis, thus elevating the levels of nuclear β-catenin, forming β-catenin/FXR complex and subsequently impairing the tumor-suppressor effect of FXR. Furthermore, due to DNA hypermethylation^30^, miRNA regulation^58^, or transcription factor regulation^59^, loss of FXR enhances the β-catenin/FXR complex and leads to persistent activation of Wnt signaling to further promote tumorigenesis (Supplementary Fig. 10). Intriguingly, Selmin et al.^60^ reported that APC mutations, which result in Wnt signaling activation, cause silencing of FXR expression through CpG hypermethylation, although the underlying mechanism remains unclear. Further investigation into the reciprocal relationship between FXR and β-catenin is urgent.

In summary, we found that FXR is downregulated in colon cancer and is negatively associated with poor prognosis. Functional studies indicated that FXR exerts a tumor-suppressive function in CRC. Mechanistically, FXR suppresses the activity of Wnt/β-catenin signaling via interaction with β-catenin. Furthermore, the FXR/β-catenin interaction retards FXR-mediated transcriptional activation of its target gene SHP. These novel findings have identified a heretofore unrecognized relationship between FXR and β-catenin in tumorigenesis, thus providing a novel interventional opportunity.

## Supplementary information

Supplementary Figures legend

Supplementary Tables

Supplementary Figure 1

Supplementary Figure 2

Supplementary Figure 3

Supplementary Figure 4

Supplementary Figure 5

Supplementary Figure 6

Supplementary Figure 7

Supplementary Figure 8

Supplementary Figure 9

Supplementary Figure 10
