# Supplementary material for: Farnesoid X receptor antagonizes Wnt/β-catenin signaling in colorectal tumorigenesis
Source: Cell Death Dis. 2020 Aug 17;11(8):640. doi: 10.1038/s41419-020-02819-w (PMC7431544; doi:10.1038/s41419-020-02819-w)
Supplement: Supplementary file 1 — Supplementary Figures legend [file 41419_2020_2819_MOESM1_ESM.docx]

**Fig. S1 FXR inhibits xenograft tumor growth.** **a, b** Tumor growth curves and mean weights shown for xenograft tumors formed by FXR-knockdown HT-29 (**a**) and Caco-2 (**b**) cells. **c, d** Tumor growth curves and mean weights shown for xenograft tumors formed by FXR-overexpressing SW480 (**c**) and HCT116 (**d**) cells. **e, f** IHC staining for Ki67 is shown in tumor xenografts of FXR-knockdown HT-29 and Caco-2 cells (**e**) or FXR-overexpressing SW480 and HCT116 cells (**f**). All data are the mean±SD of three independent experiments. **P*<0.05.

**Fig. S2 FXR inhibits the invasion and migration of colon cancer cells**. **a, c** Wound-healing assay in FXR-knockdown HT-29 and Caco-2 cells (**a**) or FXR-overexpressing SW480 and HCT116 cells (**c**) measured at 48 h. **b, d** The percentage of wound healing in FXR-knockdown cells (**b**) or FXR-overexpressing (**d**) cells. **e, f** Invasion assay in FXR-knockdown HT-29 and Caco-2 cells (**e**) or FXR-overexpressing SW480 and HCT116 cells (**f**). **g, h** The number of invasive cells in FXR-knockdown HT-29 and Caco-2 cells (**g**) or FXR-overexpressing SW480 and HCT116 cells (**h**). All data are the mean±SD of three independent experiments. **P*<0.05.

**Fig. S3 FXR inhibits EMT in colon cancer cells.** **a, b** Western blotting bands of EMT-related protein in FXR-knockdown HT-29 and Caco-2 cells (**a**) or FXR-overexpressing SW480 and HCT116 cells (**b**). **c, d** Quantitative analysis of EMT-related protein expression in FXR-knockdown HT-29 and Caco-2 cells (**c**) or FXR-overexpressing SW480 and HCT116 cells (**d**). All data are the mean±SD of three independent experiments. **P*<0.05.

**Fig. S4 Knockdown of FXR induces EMT in colon cancer cells.** Immunofluorescence (IF) staining of E-cadherin in FXR-knockdown HT-29 (**a**) and Caco-2 (**b**) cells. **c, d** IF staining of vimentin in FXR-knockdown HT-29 (**c**) and Caco-2 (**d**) cells. **e, f** Immunohistochemistry (IHC) staining of E-cadherin and vimentin in lung metastatic tumor tissues formed by FXR-knockdown HT-29 (**e**) and Caco-2 (**f**) cells and the control cells. All data are presented as the mean±SD from three independent experiments. **P* <0.05.

**Fig. S5 Blockage of the Wnt signaling by XAV-939 abolished the tumor-promoting effect of FXR knockdown**. **a, b** The effect of XAV-939 on the viability of FXR-knockdown HT-29 (**a**) and Caco-2 (**b**) cells detected by CCK8 assays. **c, d** The effect of XAV-939 on the cell cycle distribution of FXR-knockdown HT-29 (**c**) and Caco-2 (**d**) detected by FACS analysis. **e, f** The effect of XAV-939 on the invasion of FXR-knockdown HT-29 (**e**) and Caco-2 (**f**) cells detected by invasion assay. All data are the mean±SD of three independent experiments. **P*<0.05.

**Fig. S6 Blockage of the Wnt signaling by XAV-939 abolished the cell cycle- and EMT-related protein in colon cancer cells enhanced by FXR knockdown**. **a, b** The effect of XAV-939 on the levels of cell cycle- and EMT-related protein in FXR-knockdown HT-29 (**a**) and Caco-2 (**b**) cells. **c, d** Quantitative analysis of the expression of cell cycle- and EMT-related protein in FXR-knockdown HT-29 (**c**) and Caco-2 (**d**) cells. All data are the mean±SD of three independent experiments. **P*<0.05.

**Fig. S7 FXR inhibits colorectal tumorigenesis by regulating SHP expression**. **a, b** The effect of SHP overexpression (**a**) or knockdown (**b**) on the viability of FXR-knockdown HT-29 and Caco-2 cells or FXR-overexpressing SW480 and HCT116 cells detected by CCK8 assay. The effect of SHP overexpression (**c**) or knockdown (**d**) on on the cell cycle distribution of FXR-knockdown HT-29 and Caco-2 cells or FXR-overexpressing SW480 and HCT116 cells detected by FACS analysis. **e, f** The effect of SHP overexpression (**e**) or knockdown (**f**) on invasion of FXR-knockdown HT-29 and Caco-2 cells or FXR-overexpressing SW480 and HCT116 cells detected by invasion assay. All data are the mean±SD of three independent experiments. **P*<0.05.

**Fig. S8 The effect of modulating β-catenin on SHP expression in colon cancer cells.** **a, b** The effect of β-catenin knockdown (**a**) or β-catenin overexpression (**b**) on SHP mRNA levels in FXR-overexpressing SW480 and HCT116 cells detected by real-time PCR. **c, d** The effect of β-catenin knockdown (**c**) or β-catenin overexpression (**d**) on SHP protein levels in FXR-overexpressing SW480 and HCT116 cells detected by western blotting analysis. All data are the mean±SD of three independent experiments. **P*<0.05.

**Fig. S9** **Correlation analysis between the expression of FXR and cell cycle- and EMT-related protein in human colon cancer specimens**. **a**. Immunohistochemical staining showing FXR, SHP, E-cadherin, vimentin, cyclin D1 and c-Myc expression in colon cancer tissues. **b, c, d, e, f** Correlation of the FXR staining and the SHP (**b**, r=0.6935; P=0.0008), E-cadherin (**c**, r=0.4597; P=0.011), vimentin (**d**, r=-0.4188; P=0.0212), cyclin D1 (**e**, r=-0.5891; P=0.0006) and (**f**, r=-0.3936; P=0.0315) staining.

**Fig. S10 Schematic representation of the molecular mechanisms underlying the inhibitory effect of FXR on the proliferation and metastasis of colon cancer cells.**

In Wnt signaling inactive state, β-catenin is phosphorylated by GSK3β, ubiquitinated by β-TrCP and targeted for proteasomal degradation (**a**). At the early phase of colorectal tumorigenesis, an event initiated in tumor cells activates Wnt signaling, thus elevating the levels of nuclear β-catenin, forming β-catenin/FXR complex and subsequently impairing the tumor-suppressor effect of FXR (**b**). Furthermore, loss of FXR reduces the β-catenin/FXR complex and leads to persistent activation of Wnt signaling to further promote tumorigenesis (**c**).
