# Supplementary material for: Farnesoid X receptor antagonizes Wnt/β-catenin signaling in colorectal tumorigenesis
Source: Cell Death Dis. 2020 Aug 17;11(8):640. doi: 10.1038/s41419-020-02819-w (PMC7431544; doi:10.1038/s41419-020-02819-w)
Supplement: Supplementary file 2 — Supplementary Tables [file 41419_2020_2819_MOESM2_ESM.docx]

Table S1 Primer sequence

| Gene | Sequence |
| --- | --- |
| qRT-PCR |  |
| cyclin D1 | F: 5’- AAACAGATCATCCGCAAACAC-3’ |
|  | R: 5’-GTTGGGGCTCCTCAGGTTC-3’ |
| c-Myc | F: 5’-CCTGGTGCTCCATGAGGAGA-3’ |
|  | R:5’-TCCAGCAGAAGGTGATCCAGAC-3’ |
| SHP | F: 5’-CGATCCTCTTCAACCCAGATG-3’ |
|  | R: 5’-AGGGCTCCAAGACTTCACACA-3’ |
| GAPDH | F: 5’-TGCACCACCAACTGCTTAGC-3’ |
|  | R: 5’-GGCATGGACTGTGGTCATGAG-3’ |
| Luciferase Assays |  |
| SHP promoter | F: 5’-TCCTGCAGGGGGCCCAGAAC-3’  R: 5’-CCAGCAACCCTGCAGCAGCC-3’ |
| ChIP-qPCR |  |
| FXR/RXRα | F 5’-TGCCCTGGTACAGCCTGAGT-3’ |
|  | R 5’-ACACAGACATTGCCCCTGGC-3’ |

Table S2 Antibodies

| Antibodies | Source | | Identifier |
| --- | --- | --- | --- |
| GAPDH | | Santa Cruz | Cat#sc-47724 |
| FXR | | Santa Cruz | Cat#sc-25309 |
| vimentin | | Abcam | Cat#ab92547 |
| MMP9 | | Abcam | Cat#ab76003 |
| Snail | | Abcam | Cat#ab53519 |
| Slug | | Abcam | Cat#ab27568 |
| fibronectin | | Abcam | Cat#ab32419 |
| SHP | | Abcam | Cat#ab96605 |
| TCF4 | | Abcam | Cat#ab217668 |
| RXRα | | Cell Signaling Technology | Cat#3085 |
| E-cadherin | | Cell Signaling Technology | Cat#14472 |
| cyclin D1 | | Cell Signaling Technology | Cat#55506 |
| c-Myc | | Cell Signaling Technology | Cat#18583 |
| ZO-1 | | Cell Signaling Technology | Cat#13663 |
| β-catenin | | Cell Signaling Technology | Cat#8480 |
| Histone H3 | | Cell Signaling Technology | Cat#4499 |

| Table S3 Correlation between the factors and clinicopathologic characteristics in colon cancer | | | | |
| --- | --- | --- | --- | --- |
| Clinicopathologic indexes | FXR | | χ² | P value |
|  | Negative | Positive |  |  |
| Gender |  |  | 0.126 | 0.723 |
| Male | 47 | 24 |  |  |
| Female | 36 | 16 |  |  |
| Age in diagnosis(years) |  |  | 1.883 | 0.170 |
| ≤55 | 23 | 16 |  |  |
| >55 | 60 | 24 |  |  |
| Differentiation |  |  | 0.727 | 0.394 |
| Moderate | 70 | 36 |  |  |
| Low | 13 | 4 |  |  |
| Tumor size |  |  | 9.394 | 0.002^*^ |
| ≤5cm | 50 | 35 |  |  |
| >5cm | 33 | 5 |  |  |
| T stages |  |  | 17.167 | <0.001^*^ |
| T1-T2 | 11 | 19 |  |  |
| T3-T4 | 72 | 21 |  |  |
| Lymphatic metastasis |  |  | 16.910 | <0.001^*^ |
| No | 46 | 37 |  |  |
| Yes | 37 | 3 |  |  |
| M stages |  |  |  | 0.162^#^ |
| No | 70 | 38 |  |  |
| Yes | 13 | 2 |  |  |
| TNM stages |  |  | 16.910 | <0.001^*^ |
| Ⅰ+Ⅱ | 46 | 37 |  |  |
| Ⅲ+Ⅳ | 37 | 3 |  |  |
| Location |  |  | 0.003 | 0.955 |
| Right | 57 | 26 |  |  |
| Left | 30 | 14 |  |  |

^#^: Yates’ continuity corrected chi-square test

*P<0.05

Table S4 Cox proportional Hazard regression analysis of patients’ overall survival.

| Varibles | Univariable | | | |  | Multivariable | | | |
| --- | --- | --- | --- | --- | --- | --- | --- | --- | --- |
|  |  | 95.0% CI | |  |  |  | 95.0% CI | |  |
|  | HR | lower | upper | P |  | HR | lower | Upper | P |
| Age  (>55 vs ≤55 ) | 0.870 | 0.512 | 1.479 | 0.608 |  |  |  |  |  |
| Gender  (Male vs Female ) | 1.076 | 0.653 | 1.733 | 0.773 |  |  |  |  |  |
| Differentiation  (Low vs Moderate ) | 0.332 | 0.117 | 0.585 | <0.001^*^ |  | 0.444 | 0.234 | 0.840 | 0.013^*^ |
| Tumor size  (>5cm vs ≤5cm) | 1.896 | 1.139 | 3.158 | 0.014^*^ |  | 1.349 | 0.729 | 2.496 | 0.341 |
| T stages  (T3-T4 vs T1-T2) | 2.642 | 1.302 | 5.358 | 0.007^*^ |  | 1.803 | 0.818 | 3.976 | 0.144 |
| Lymphatic metastasis  (Yes vs No) | 7.361 | 4.370 | 12.399 | <0.001^*^ |  | 5.643 | 2.869 | 11.102 | <0.001^*^ |
| M stages  (Yes vs No) | 8.134 | 4.039 | 15.356 | <0.001^*^ |  | 1.818 | 0.821 | 4.025 | 0.141 |
| TNM stages  (Ⅲ+ⅣvsⅠ+Ⅱ) | 7.361 | 4.370 | 12.399 | <0.001^*^ |  | NA |  |  |  |
| Location  （Right vs Left） | 0.973 | 0.589 | 1.607 | 0.915 |  |  |  |  |  |
| FXR expression  (High vs Low) | 0.471 | 0.260 | 0.854 | 0.013^*^ |  | 1.355 | 0.645 | 2.847 | 0.423 |

NA: TNM stages= Lymphatic metastasis

Table S5 Cox proportional Hazard regression analysis of patients’ recurrence-free survival.

| Varibles | Univariable | | | |  | Multivariable | | | |
| --- | --- | --- | --- | --- | --- | --- | --- | --- | --- |
|  |  | 95.0% CI | |  |  |  | 95.0% CI | |  |
|  | HR | lower | upper | P |  | HR | lower | Upper | P |
| Age  (>55 vs ≤55 ) | 0.932 | 0.573 | 1.516 | 0.777 |  |  |  |  |  |
| Gender  (Male vs Female ) | 1.292 | 0.828 | 2.015 | 0.259 |  |  |  |  |  |
| Differentiation  (Low vs Moderate ) | 0.328 | 0.183 | 0.591 | <0.001^*^ |  | 0.500 | 0.264 | 0.948 | 0.034^*^ |
| Tumor size  (>5cm vs ≤5cm) | 1.282 | 0.798 | 2.061 | 0.305 |  |  |  |  |  |
| T stages  (T3-T4 vs T1-T2) | 2.661 | 1.405 | 5.037 | 0.003^*^ |  | 2.069 | 1.050 | 4.076 | 0.036^*^ |
| Lymphatic metastasis  (Yes vs No) | 3.782 | 2.356 | 6.072 | <0.001^*^ |  | 2.361 | 1.308 | 4.262 | 0.004^*^ |
| M stages  (Yes vs No) | 4.998 | 2.695 | 9.270 | <0.001^*^ |  | 1.986 | 0.977 | 4.037 | 0.058 |
| TNM stages  (Ⅲ+ⅣvsⅠ+Ⅱ) | 3.782 | 2.356 | 6.072 | <0.001^*^ |  |  |  |  |  |
| Location  （Right vs Left） | 0.733 | 0.466 | 1.152 | 0.179 |  |  |  |  |  |
| FXR expression  (High vs Low) | 0.542 | 0.322 | 0.910 | 0.020^*^ |  | 0.927 | 0.519 | 1.657 | 0.798 |

NA: TNM stages= Lymphatic metastasis
