# Supplementary figures and images for: Farnesoid X receptor antagonizes Wnt/β-catenin signaling in colorectal tumorigenesis
Source: Cell Death Dis. 2020 Aug 17;11(8):640. doi: 10.1038/s41419-020-02819-w (PMC7431544; doi:10.1038/s41419-020-02819-w)

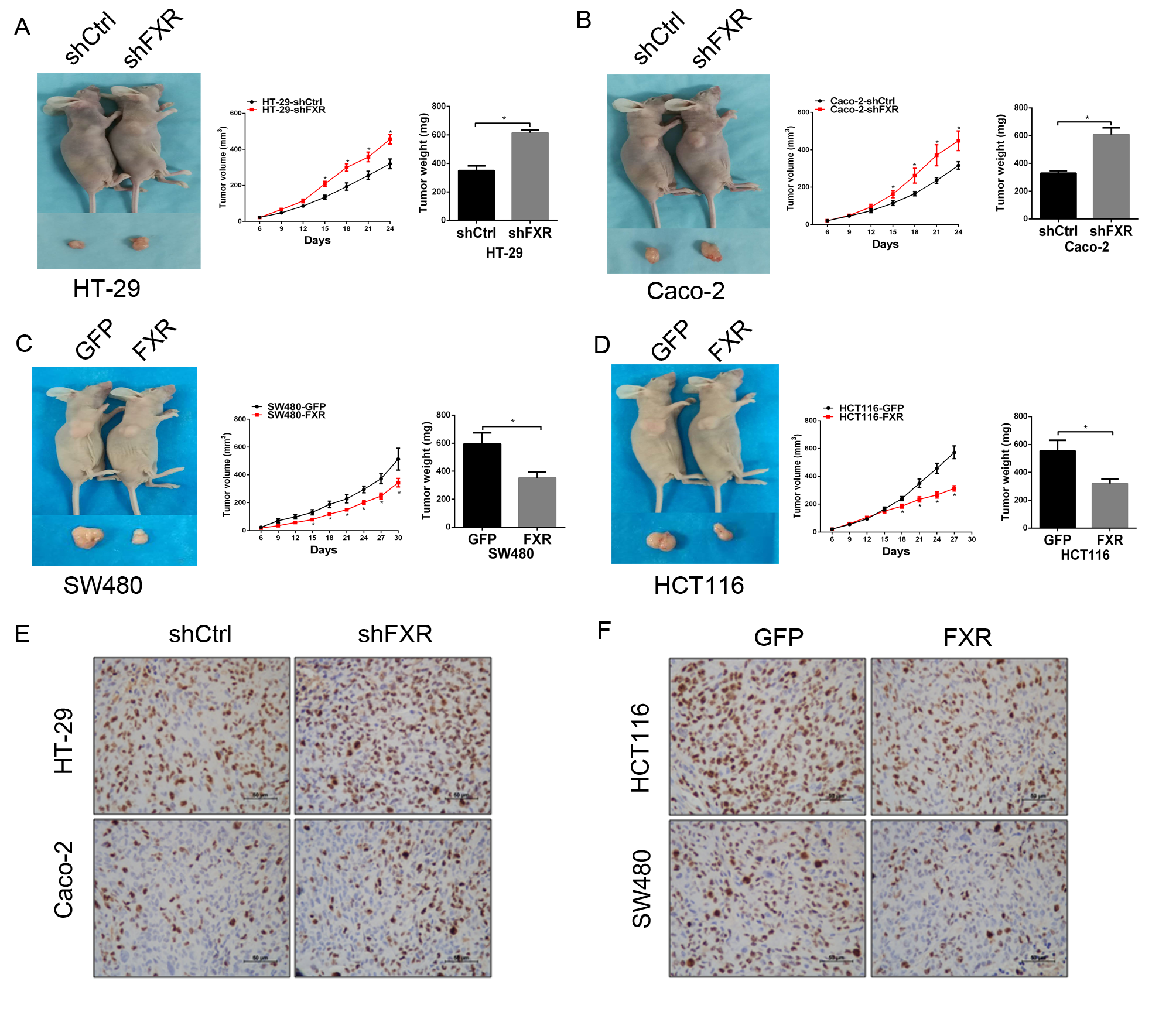

Supplement: Supplementary file 3 — Supplementary Figure 1 [file 41419_2020_2819_MOESM3_ESM.tif]

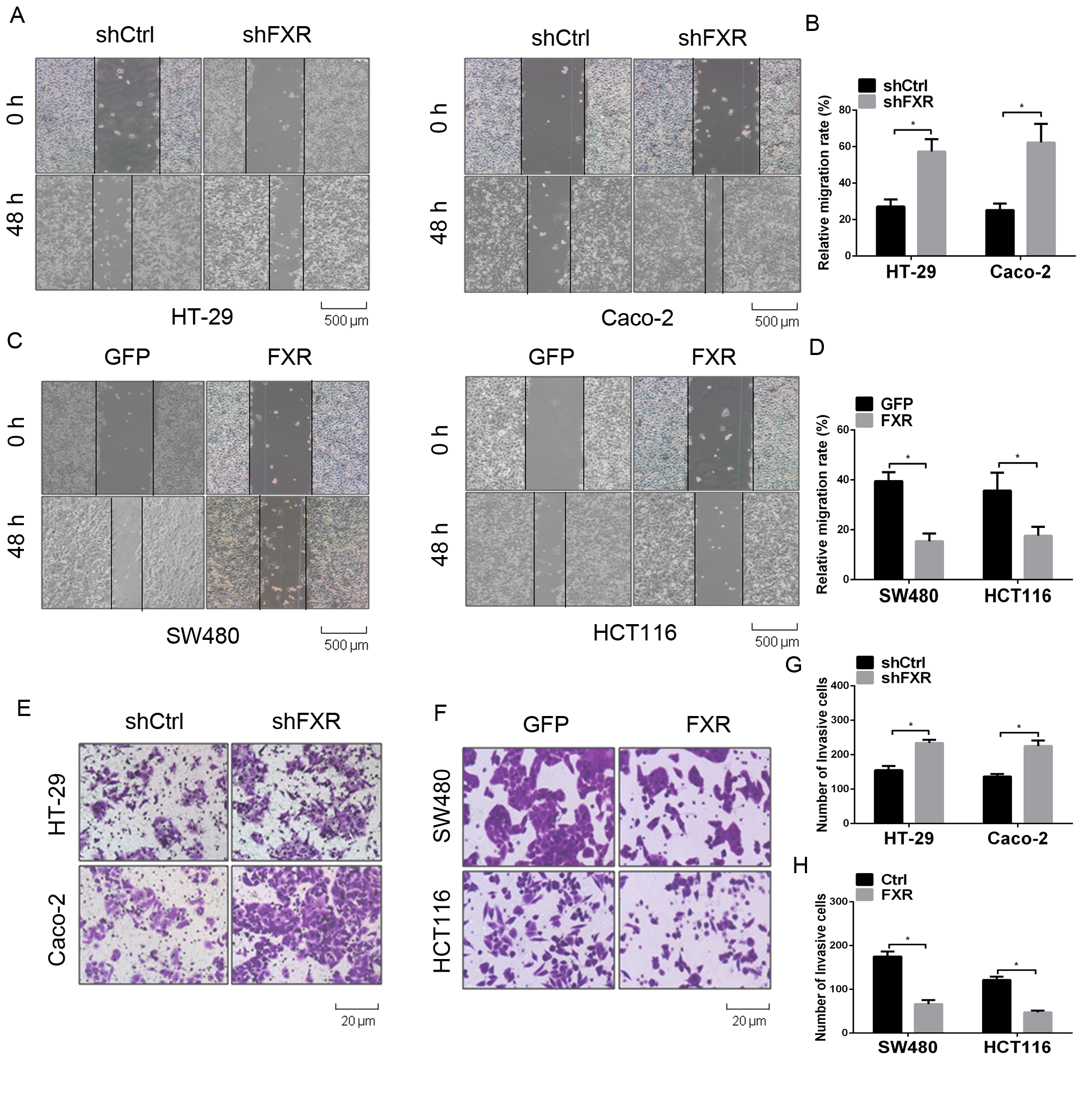

Supplement: Supplementary file 4 — Supplementary Figure 2 [file 41419_2020_2819_MOESM4_ESM.tif]

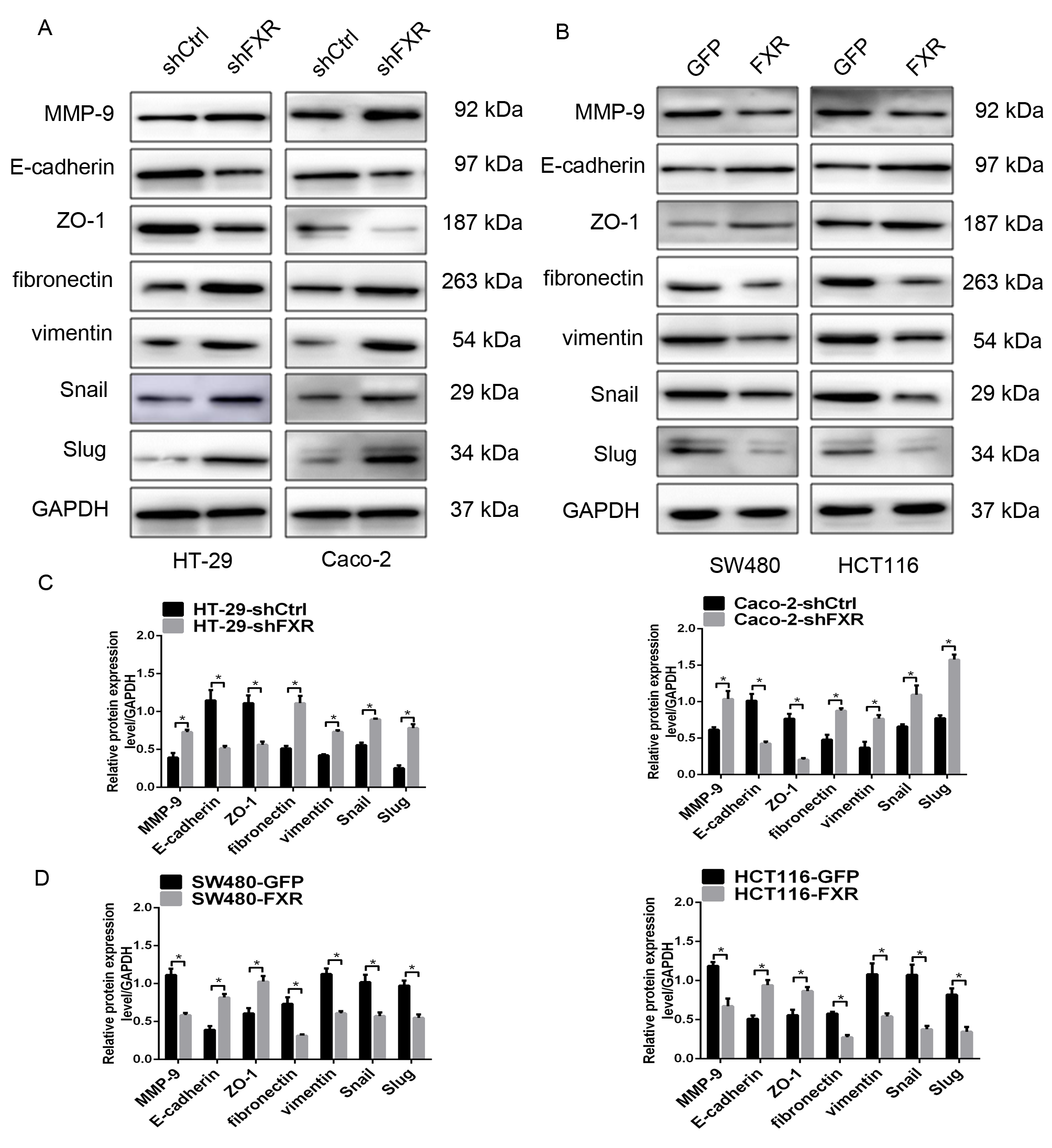

Supplement: Supplementary file 5 — Supplementary Figure 3 [file 41419_2020_2819_MOESM5_ESM.tif]

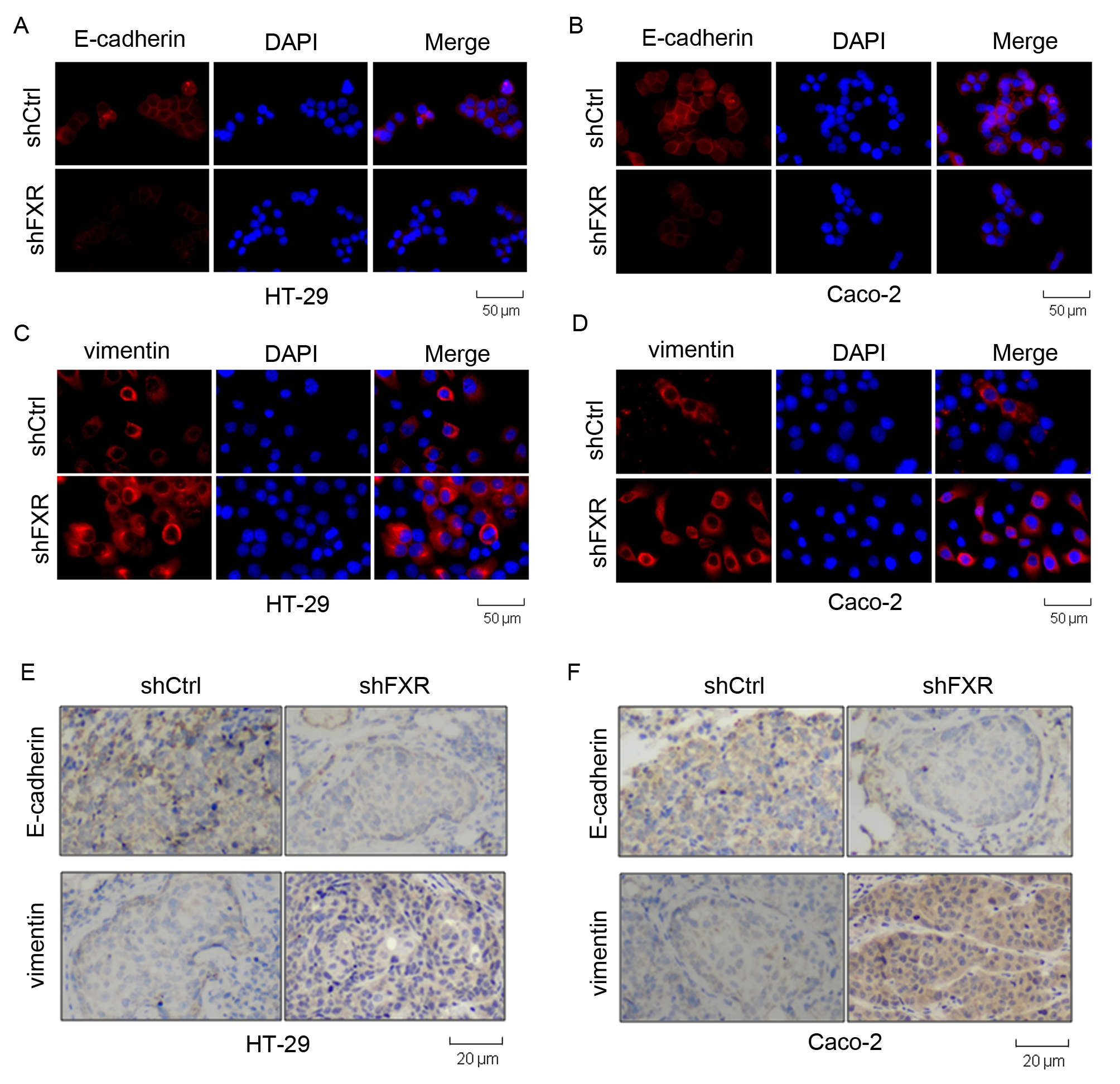

Supplement: Supplementary file 6 — Supplementary Figure 4 [file 41419_2020_2819_MOESM6_ESM.tif]

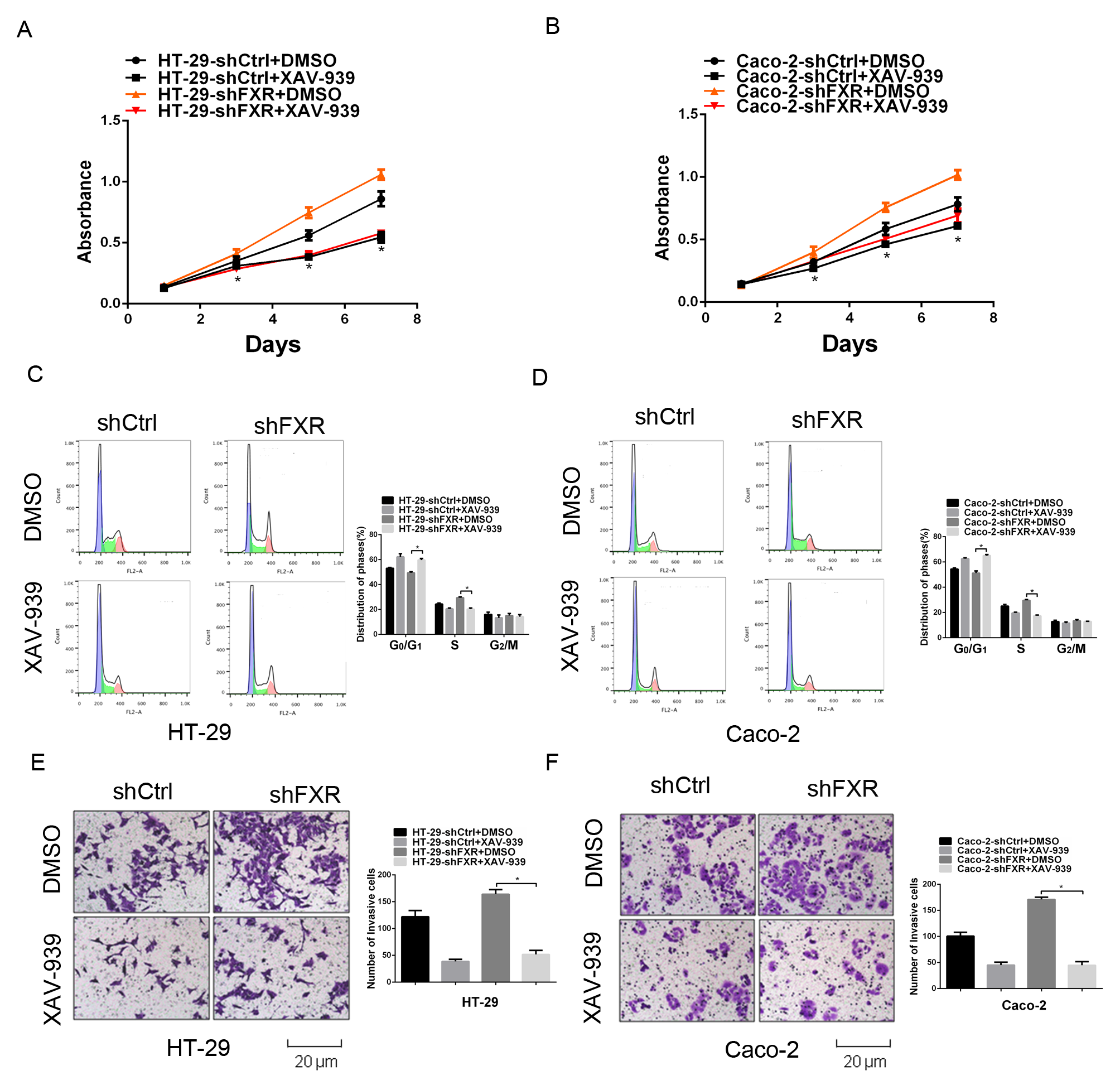

Supplement: Supplementary file 7 — Supplementary Figure 5 [file 41419_2020_2819_MOESM7_ESM.tif]

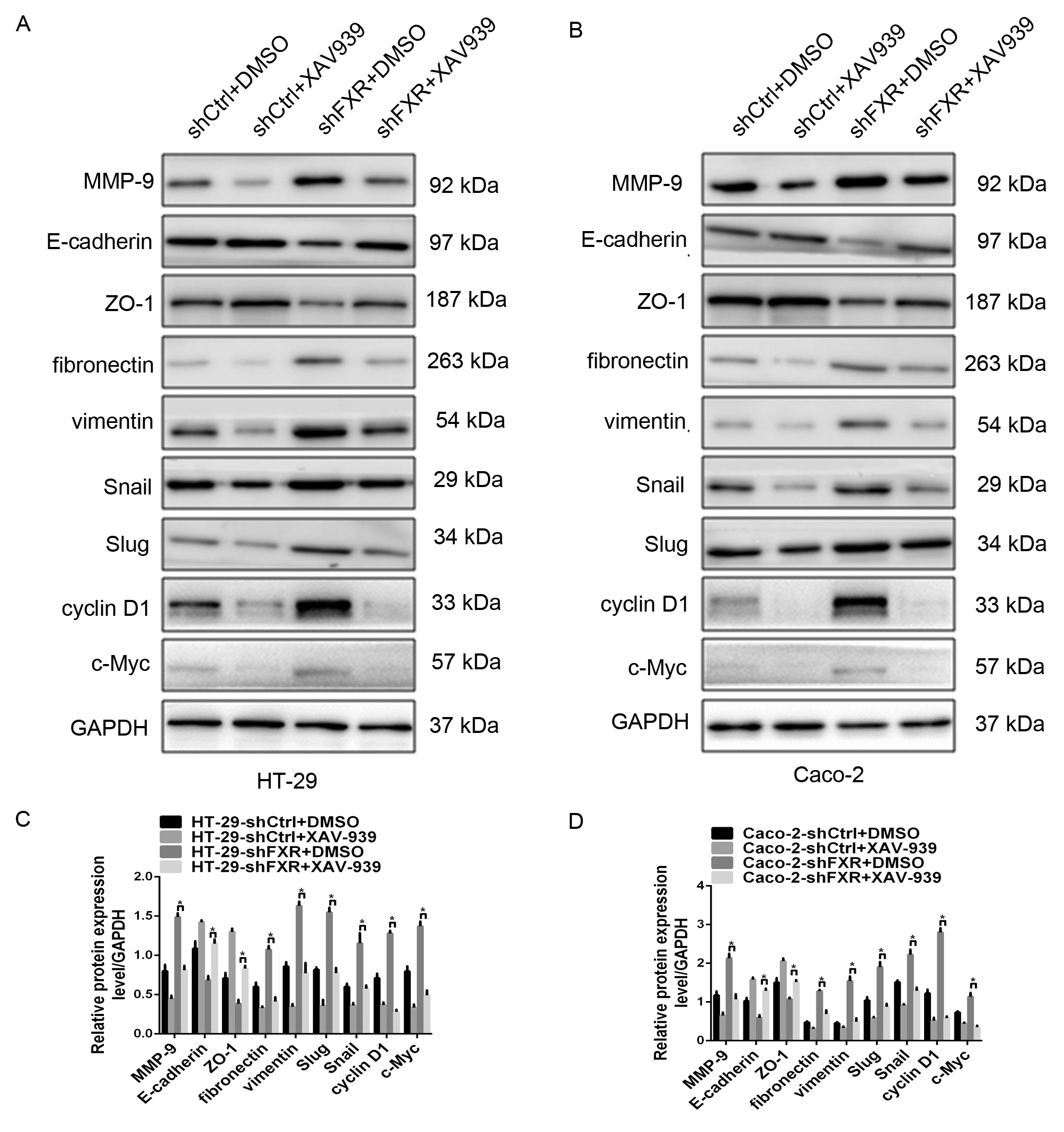

Supplement: Supplementary file 8 — Supplementary Figure 6 [file 41419_2020_2819_MOESM8_ESM.tif]

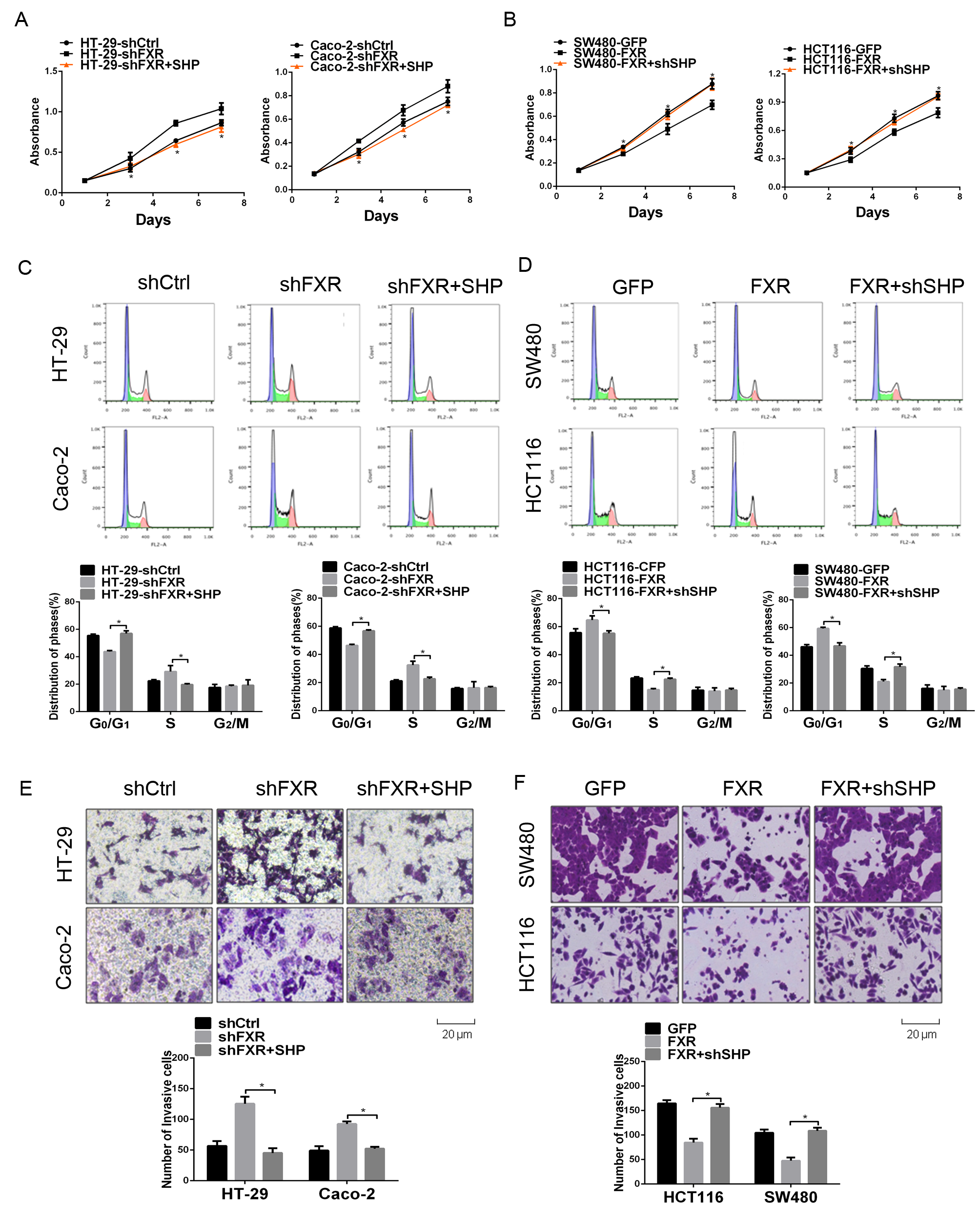

Supplement: Supplementary file 9 — Supplementary Figure 7 [file 41419_2020_2819_MOESM9_ESM.tif]

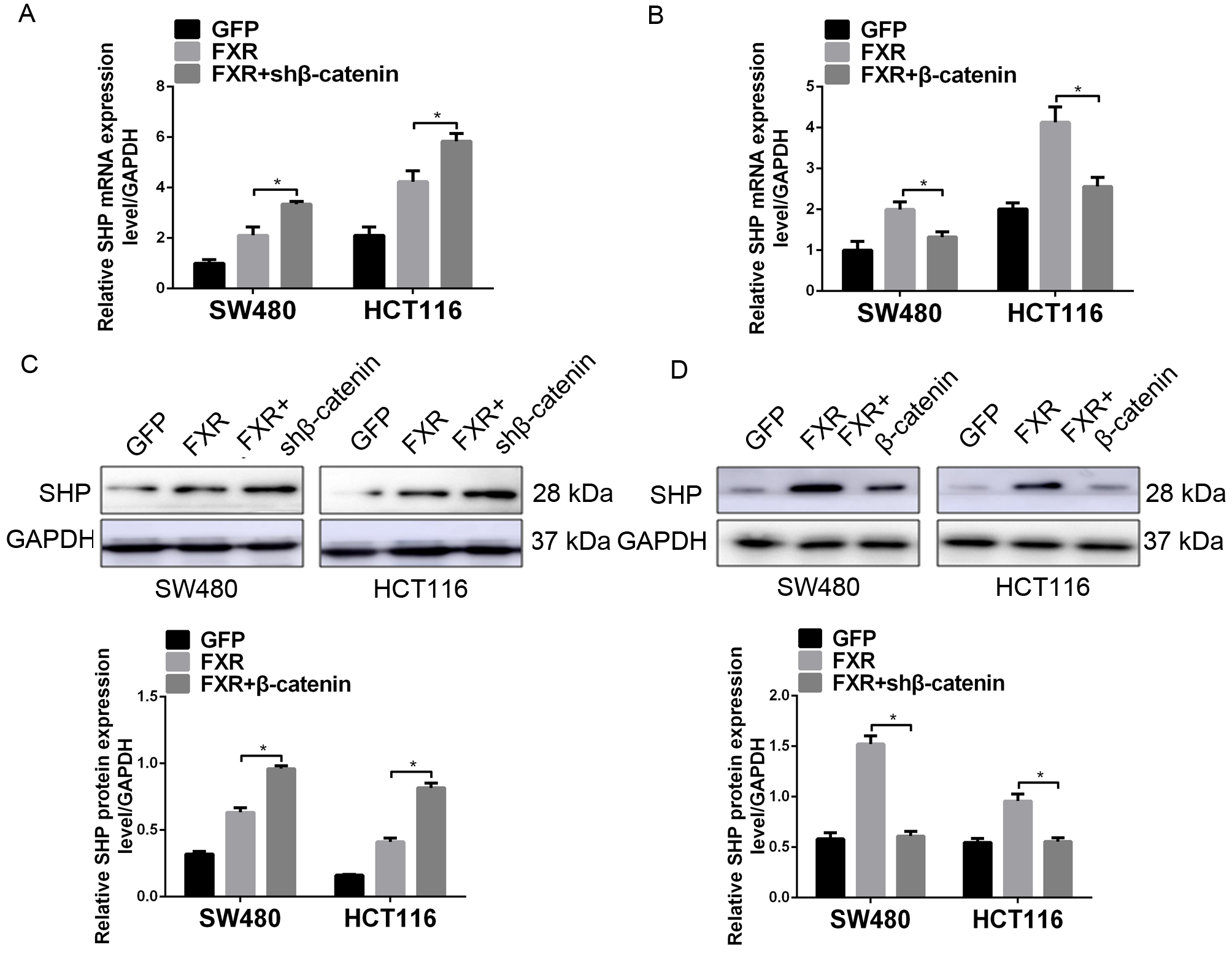

Supplement: Supplementary file 10 — Supplementary Figure 8 [file 41419_2020_2819_MOESM10_ESM.tif]

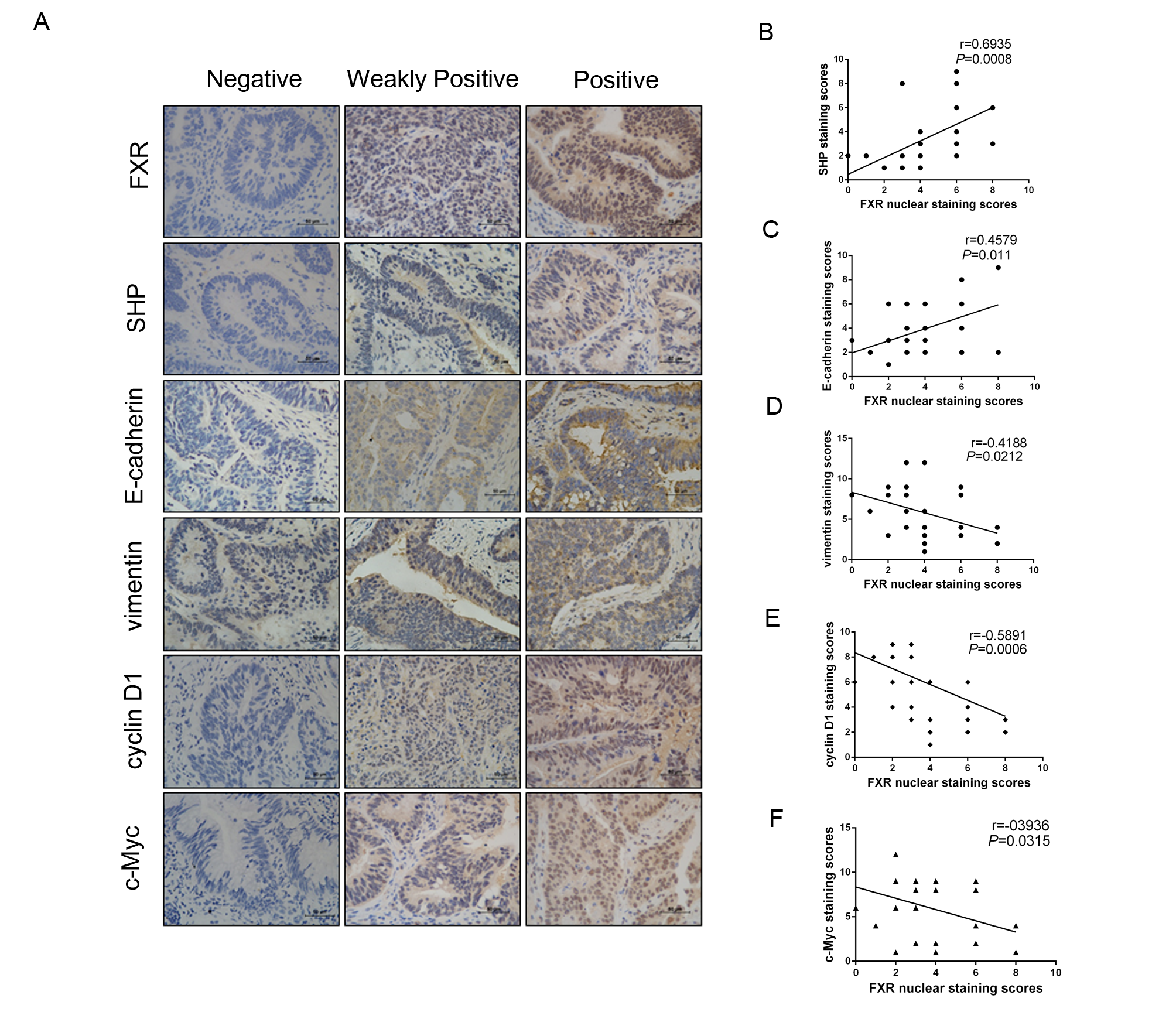

Supplement: Supplementary file 11 — Supplementary Figure 9 [file 41419_2020_2819_MOESM11_ESM.tif]

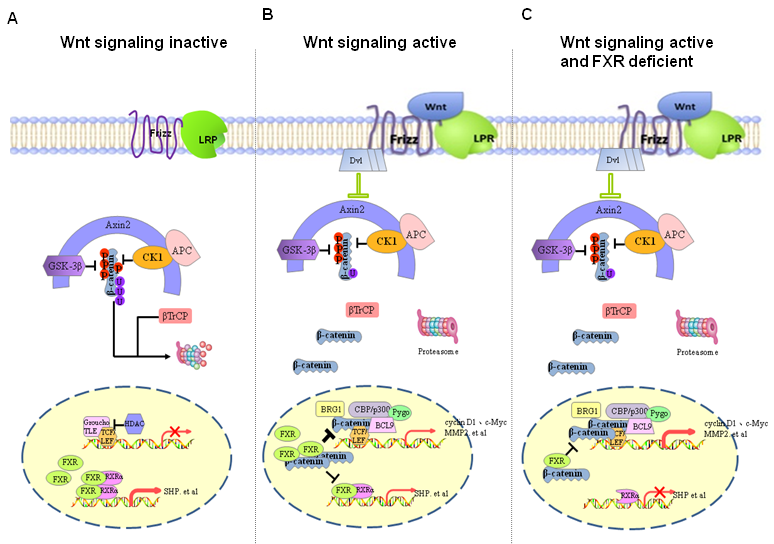

Supplement: Supplementary file 12 — Supplementary Figure 10 [file 41419_2020_2819_MOESM12_ESM.tif]
